# Physiology and Evolution of Voltage-Gated Calcium Channels in Early Diverging Animal Phyla: Cnidaria, Placozoa, Porifera and Ctenophora

**DOI:** 10.3389/fphys.2016.00481

**Published:** 2016-11-04

**Authors:** Adriano Senatore, Hamad Raiss, Phuong Le

**Affiliations:** Department of Biology, University of Toronto MississaugaMississauga, ON, Canada

**Keywords:** calcium channel evolution, pre-synaptic exocytosis, excitation-contracting coupling, regulation of ciliary beating, synaptic scaffolding, early-diverging animals, evolution of the nervous system, synapse evolution

## Abstract

Voltage-gated calcium (Ca_v_) channels serve dual roles in the cell, where they can both depolarize the membrane potential for electrical excitability, and activate transient cytoplasmic Ca^2+^ signals. In animals, Ca_v_ channels play crucial roles including driving muscle contraction (excitation-contraction coupling), gene expression (excitation-transcription coupling), pre-synaptic and neuroendocrine exocytosis (excitation-secretion coupling), regulation of flagellar/ciliary beating, and regulation of cellular excitability, either directly or through modulation of other Ca^2+^-sensitive ion channels. In recent years, genome sequencing has provided significant insights into the molecular evolution of Ca_v_ channels. Furthermore, expanded gene datasets have permitted improved inference of the species phylogeny at the base of Metazoa, providing clearer insights into the evolution of complex animal traits which involve Ca_v_ channels, including the nervous system. For the various types of metazoan Ca_v_ channels, key properties that determine their cellular contribution include: Ion selectivity, pore gating, and, importantly, cytoplasmic protein-protein interactions that direct sub-cellular localization and functional complexing. It is unclear when these defining features, many of which are essential for nervous system function, evolved. In this review, we highlight some experimental observations that implicate Ca_v_ channels in the physiology and behavior of the most early-diverging animals from the phyla Cnidaria, Placozoa, Porifera, and Ctenophora. Given our limited understanding of the molecular biology of Ca_v_ channels in these basal animal lineages, we infer insights from better-studied vertebrate and invertebrate animals. We also highlight some apparently conserved cellular functions of Ca_v_ channels, which might have emerged very early on during metazoan evolution, or perhaps predated it.

## Introduction

The coupling of fast electrical impulses, driven by voltage-gated potassium (K_v_) and sodium (Na_v_) channels, with calcium-dependent synaptic signaling, allows the nervous system to coordinate cellular activities rapidly and over long distances. In the pre-synaptic terminal of neurons, electrical impulses trigger secretion of neurotransmitters via the action of voltage-gated Ca^2+^ (Ca_v_) channels, positioned within nanometers of the Ca^2+^-sensitive exocytotic machinery. This proximity serves to overcome the strong sequestration and extrusion of Ca^2+^ from the cytosol in response to the ion's intracellular toxicity (Clapham, 2007; Stanley, 2016). The Ca^2+^ ion distinguishes itself from the more abundant K^+^ and Na^+^ by its ability to strongly bind oxygen-bearing proteins, altering their conformation. Ca_v_ channels are thus able to convert electrical signals carried by K_v_ and Na_v_ channels into cytoplasmic Ca^2+^ signals, which can be “local,” situated in close proximity to the channel pore, or “global,” relayed by soluble Ca^2+^-activated second messengers such as calmodulin (Ikeda, 2001; Clapham, 2007). Local processes controlled by Ca_v_ channels and directly by Ca^2+^ include exocytosis, modulation/activation of other ion channels [e.g., ryanodine receptors (Lanner et al., 2010), BK and SK potassium channels (Vergara et al., 1998; Stocker, 2004), chloride channels (Berg et al., 2012)], regulation of ciliary/flagellar beating (Tamm, 1994, 2014a; Fujiu et al., 2009), and contraction of various muscle cell types (Bers, 2002). Global effects mediated by Ca_v_ channels include changes in gene expression associated with learning and memory (Dolmetsch, 2003), control of cell proliferation (Lory et al., 2006; Taylor et al., 2008; Monteith et al., 2012; Borowiec et al., 2014), and control of neurite outgrowth (Lory et al., 2006).

Extensive research has been carried out to understand Ca_v_ channel physiology and pathology (Perez-Reyes, 2003; Catterall, 2011; Dolphin, 2013; Simms and Zamponi, 2014), relying largely on genetic association studies in human disease and select experimental model species including mouse, rat, *Drosophila, C.elegans*, and a few others. Beyond vertebrate and invertebrate animal model systems, however, we have a limited understanding of the roles that specific Ca_v_ channels play, especially in the most early diverging lineages. Although numerous endogenous voltage-gated calcium channel currents have been recorded from tissue/cellular preparations derived from these basal animals, little is known about the underlying molecular biology and its homology to better understood systems. Below, we review what is known about voltage-gated calcium channel physiology in the basal animal phyla of Cnidaria, Placozoa, Porifera, and Ctenophora, in light of a clearer genomic identity of their Ca_v_ channels. We also attempt to link experimental observations of voltage-gated Ca^2+^ channel activity and localization in these animals with inferred distinguishing features of the different Ca_v_ channel types as defined in well-studied animal systems. By extension, we discuss seemingly conserved aspects of Ca_v_ channel function that might have emerged very early on during evolution of the nervous system, or perhaps predated it.

## Phylogenetic relationships of early-diverging animals

**Figure 1:**
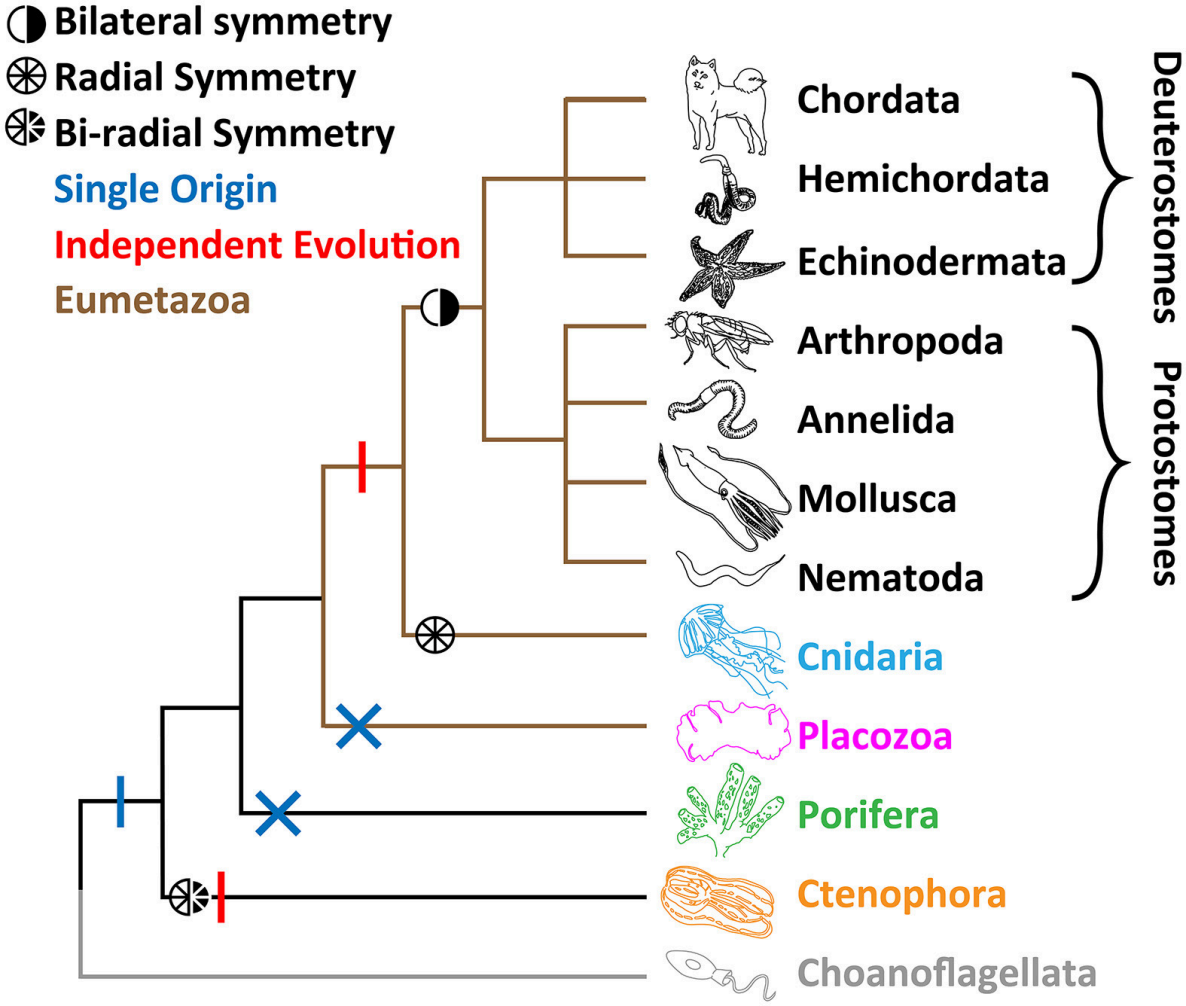
**Leading phylogeny of early-diverging animal phyla**. The two alternate hypotheses for nervous system are depicted. The single origin hypothesis involves emergence at the stem of Metazoa (blue vertical bar), and losses in both Porifera (sponges) and Placozoa (*Trichoplax*; blue crosses), whereas the independent origins hypotheses involves separate emergence in Ctenophora (comb jellies) vs. Cnidaria (jellyfish, sea anemones, corals) and Bilateria (animals with bilateral symmetry; red vertical bars).

Comparative physiology of metazoan Ca_v_ channels requires a clearly-resolved species phylogeny, which despite an emerging consensus, is still a subject of debate. Metazoans are divided into four major groups based on body symmetry (Figure 1): (1) Animals with bilateral body symmetry (bilaterians), proposed to make up over 99% of all animal species (Finnerty et al., 2004; Ryan and Chiodin, 2015), further subdivided into Deuterostomia (e.g., phyla Chordata, Hemichordata, and Echinodermata) and Protostomia (e.g., Arthropoda, Nematoda, Mollusca, and Annelida) (Wray, 2015); (2) Animals with radial body symmetry, from the phylum Cnidaria (e.g., jellyfish, corals, sea anemones, and hydra); (3) Animals that lack body symmetry, from the phyla Porifera (sponges) and Placozoa (*Trichoplax* sp.); and (4) Animals with bi-radial body symmetry, from the phylum Ctenophora (i.e., comb-jellies), which have a combination of bilateral and radial symmetry (Tamm, 2014a).

Until relatively recently, Porifera were broadly thought to sit at the base of Metazoa, due to their morphological simplicity, lack of nervous systems and musculature, and their bearing choanocytes, flagellated cells with a striking resemblance to single-celled choanoflagellates (Kent, 1880; Leadbeater and Kelly, 2001; King, 2004; King et al., 2008). Placozoans, amoeba-like benthic sea creatures (Eitel et al., 2013) which like sponges lack body symmetry, neurons, synapses and muscle, were also ascribed to this basal position (Schulze, 1892; Schierwater, 2005; Schierwater et al., 2009). A phylogenetic analysis of mitochondrial genomes supported *Trichoplax* as the basal metazoan (Dellaporta et al., 2006), however, subsequent expansive phylogenetic efforts, using nuclear encoded genes in the sequenced genomes of both *Trichoplax* and the sponge *Amphimedon queenslandica*, pointed to Porifera as the sister group to all animals, and Placozoa as a sister group to Cnidaria and Bilateria, forming the clade Eumetazoa or “true” animals (Srivastava et al., 2008, 2010) (Figure 1). The apparent resolution was not long lasting however, beginning in 2008 with a large-scale phylogenetic analysis, using expansive expressed sequence tag (EST) data, suggesting ctenophores are the most early-diverging animals (Dunn et al., 2008). This notion incited a debate about the origin of the nervous system (Marlow and Arendt, 2014; Halanych, 2015; Jékely et al., 2015; Ryan and Chiodin, 2015; Moroz and Kohn, 2016). Under the “ctenophore first” phylogeny, the absence of nervous systems in Porifera and Placozoa would suggest that the last common ancestor of all animals had a nervous system, and that these two phyla lost it. Alternatively, and more controversially, ctenophores independently evolved synapses and the nervous system (Figure 1). Instead, if sponges are the basal extant metazoan, the nervous system might have evolved only once, and was lost in placozoans. More recently, the genomes of two ctenophore species, *Mnemiopsis leidyi* and *Pleurobranchia bachia*, were published, both providing phylogenetic support for ctenophores as the most-early diverging group (Ryan et al., 2013; Moroz et al., 2014). These findings were corroborated in an expanded effort, where the authors sought to curtail potential systematic errors in phylogenetic inference (Whelan et al., 2015). However, an alternate analysis suggests that errors remain, and that sponges should reclaim the esteemed basal position (Pisani et al., 2015). Clearly, more work needs to be done to resolve the issue. However, it can be said with more and more certainty that either Ctenophora or Porifera are the most early-diverging extant animals, and that ctenophores possess the most divergent nervous systems in the animal Kingdom.

## Ca_v_ channel structure and molecular phylogeny

Our foray into understanding Ca_v_ channel molecular identity, structure and function began with intracellular voltage-clamp recording of various vertebrate and invertebrate tissue preparations, providing distinctions in observed Ca^2+^ currents such as voltages of activation, ion selectivity, and kinetics for activation and inactivation. One major distinction is the presence of separate low voltage activated (LVA) and high voltage activated (HVA) Ca^2+^ currents, with major implications for function since LVA channels are activated below action potential threshold, and hence serve to regulate excitability, while HVA channels are activated after action potential initiation, and hence serve as major effectors for transient Ca^2+^ signaling (Hagiwara et al., 1975; Carbone and Lux, 1984; Fedulova et al., 1985). Pharmacology with selective blockers, capable of distinguishing between different Ca^2+^ currents in recorded preparations, provided further evidence for the existence of multiple Ca_v_ channel types (Catterall et al., 2005; Dolphin, 2006). Ultimately, biochemical protein isolation, protein and gene sequencing, and phylogenetics revealed the existence of three distinct subtypes of Ca_v_ channels in animals (Ertel et al., 2000): Ca_v_1 and Ca_v_2, which conduct HVA Ca^2+^ currents, and Ca_v_3, which conduct LVA Ca^2+^ currents. As discussed below, distinct voltage-gated Ca^2+^ currents have also been recorded in preparations from early diverging animals, however, the specific ion channels involved are largely uncharacterized at the molecular level.

**Figure 2:**
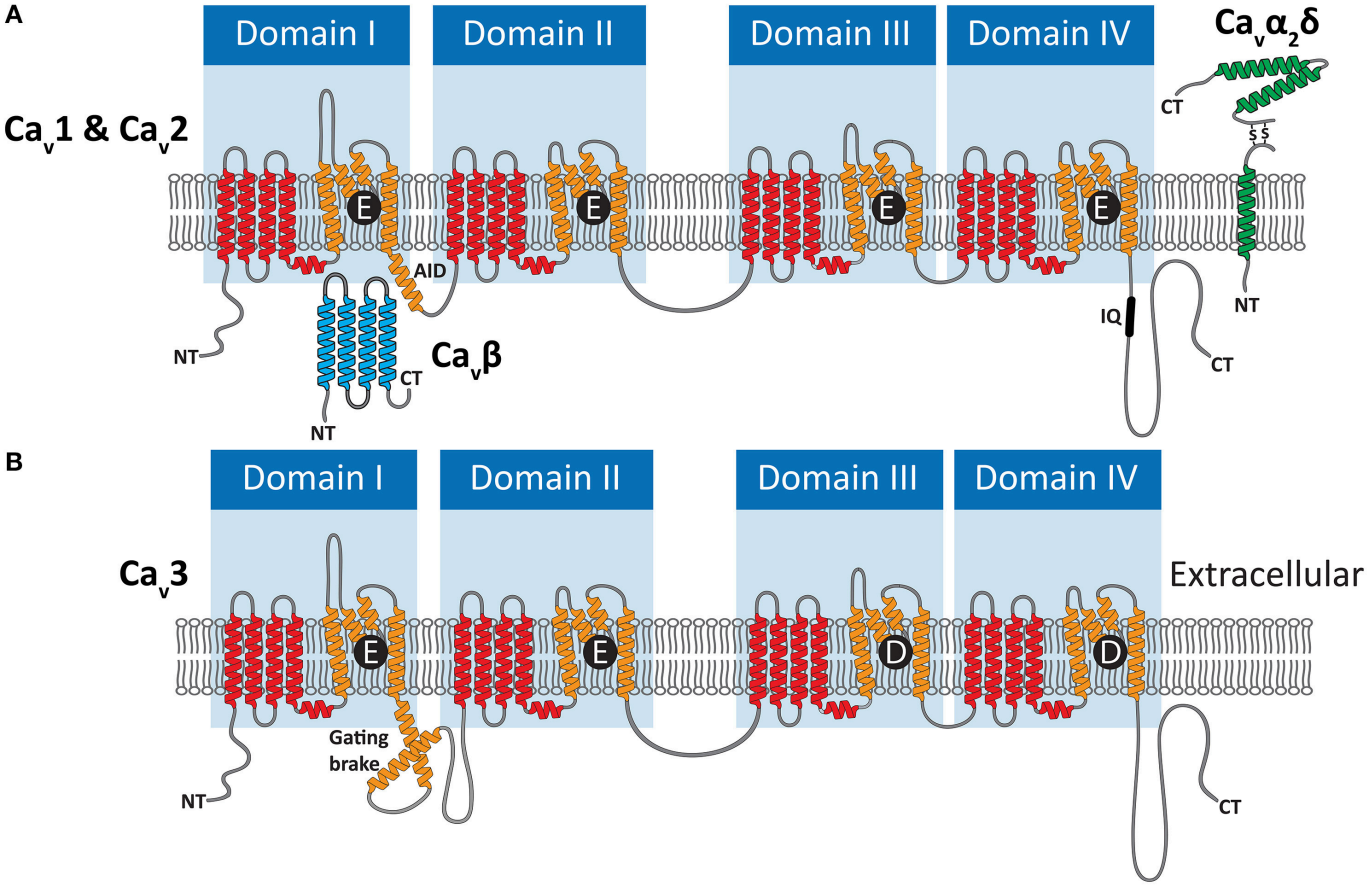
**(A)** Illustration of the membrane topology of P-loop Ca_v_1 (L-type) and Ca_v_2 (N-, P/Q-, and R-type) channels, depicting their HVA selectivity filter motifs of EEEE. Voltage sensor S1–S4 helices are colored red, and pore-forming S5 and S6 helices bearing the pore-loops orange. HVA channels interact with the cytoplasmic Ca_v_β subunit via the alpha interaction domain (AID) in the domain I-II linker, and the Ca_v_α_2_δ subunit which is anchored to the membrane and projects to the extracellular space. **(B)** Ca_v_3 (T-type channels) bear EEDD selectivity filters, do not interact with Ca_v_β and Ca_v_α_2_δ subunits, and in place of the AID bear helix-loop helix gating brake structures.

**Figure 3:**
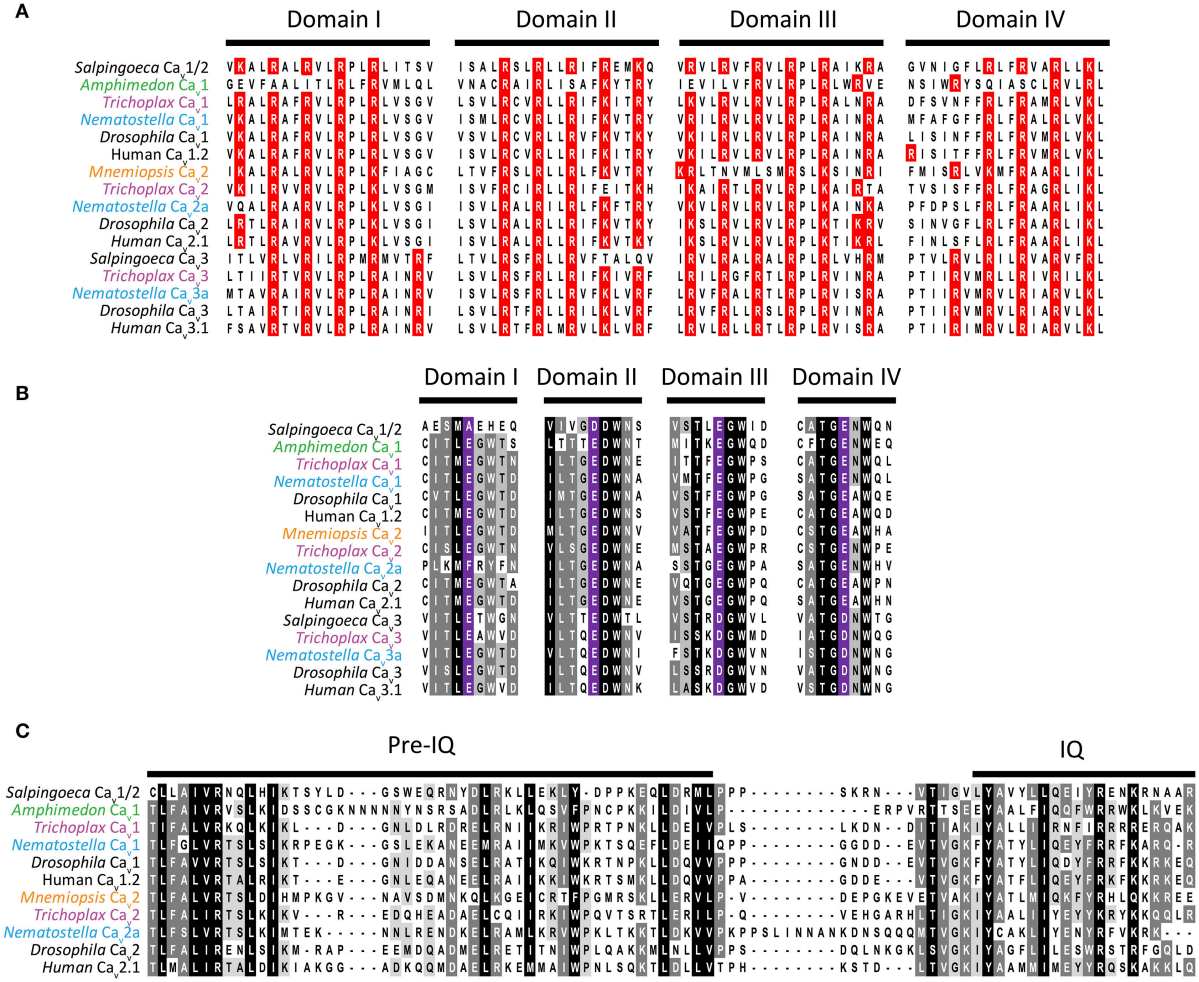
**(A)** Protein sequence alignment of domain I to IV S4 helices from Ca_v_ channel voltage sensors, depicting the strong conservation of positively charged lysine (K) and arginine (R) residues critical for voltage sensitivity. **(B)** Alignment of selectivity filter motifs and flanking amino acids from various Ca_v_ channel proteins, revealing conserved EEEE motifs for Ca_v_1, Ca_v_2, and Ca_v_1/2 channels, and EEDD for Ca_v_3 channels. **(C)** Protein sequence alignment of C-terminal IQ motifs found in Ca_v_1, Ca_v_2, and Ca_v_1/2 channel types.

Ca_v_ channels belong to a large family of cationic P-loop channels, so named because of their characteristic extracellular pore-loops that project into the narrowest part of the ion-conduction pathway to select for either Na^+^, K^+^, or Ca^2+^ ions (Mackinnon, 1995). The Ca_v_ channel structure consists of four homologous repeat domains (domains I to IV, Figure 2), each bearing 6 transmembrane alpha helices dubbed segments 1 to 6 (S1–S6). S1 to S4 helices make up the voltage sensors, with S4 helices bearing positively-charged lysine (K) and/or arginine (R) residues for sensing charge gradients across the membrane (Figure 3A) (Wu et al., 2016). Depolarization causes S4 helices to slide upwards out of the membrane to open the channel pore (Catterall, 2012). The four P-loops of metazoan Ca_v_ channels, situated between pore-forming S5 and S6 helices, contain key glutamate (E) or aspartate (D) amino acids with carboxyl oxygen atoms that form high affinity binding sites for Ca^2+^ ions, in so-called “selectivity filter” motifs, crucial for selectivity of Ca^2+^ over Na^+^ and K^+^ (Figure 2). Structurally, HVA and LVA channels bear three major differences: (1) Ca_v_1 and Ca_v_2 channel selectivity filters are comprised of four glutamate residues (i.e., selectivity filter motifs of EEEE), whereas Ca_v_3 channel filters are comprised of two glutamates and two aspartates (EEDD) (Figures 2, 3B); (2) the intracellular cytoplasmic linker between Domains I and II of Ca_v_1 and Ca_v_2 channels bear a rigid alpha helix, termed the alpha-interaction domain (AID), where accessory cytoplasmic subunit Ca_v_β binds to and regulates the channels (Figure 2A) (Wu et al., 2016), while in this equivalent position, Ca_v_3 channels bear a conserved helix-loop-helix motif, dubbed the gating brake (Perez-Reyes, 2010), which plays an important role in low voltage gating (Figure 2B); and (3) Ca_v_1 and Ca_v_2 channels bear conserved isoleucine-glutamine (IQ) motifs in their C-termini, absent in Ca_v_3 channels, which mediate physical coupling with the cytoplasmic Ca^2+^ sensor calmodulin (Figures 2A, 3C). Activation of calmodulin by channel opening and elevated cytoplasmic [Ca^2+^] imposes conformational changes in the channel structure leading to more rapid transition to non-conducting inactivated states, limiting the amount of Ca^2+^ that enters the cell upon membrane depolarization (Simms and Zamponi, 2014).

**Figure 4:**
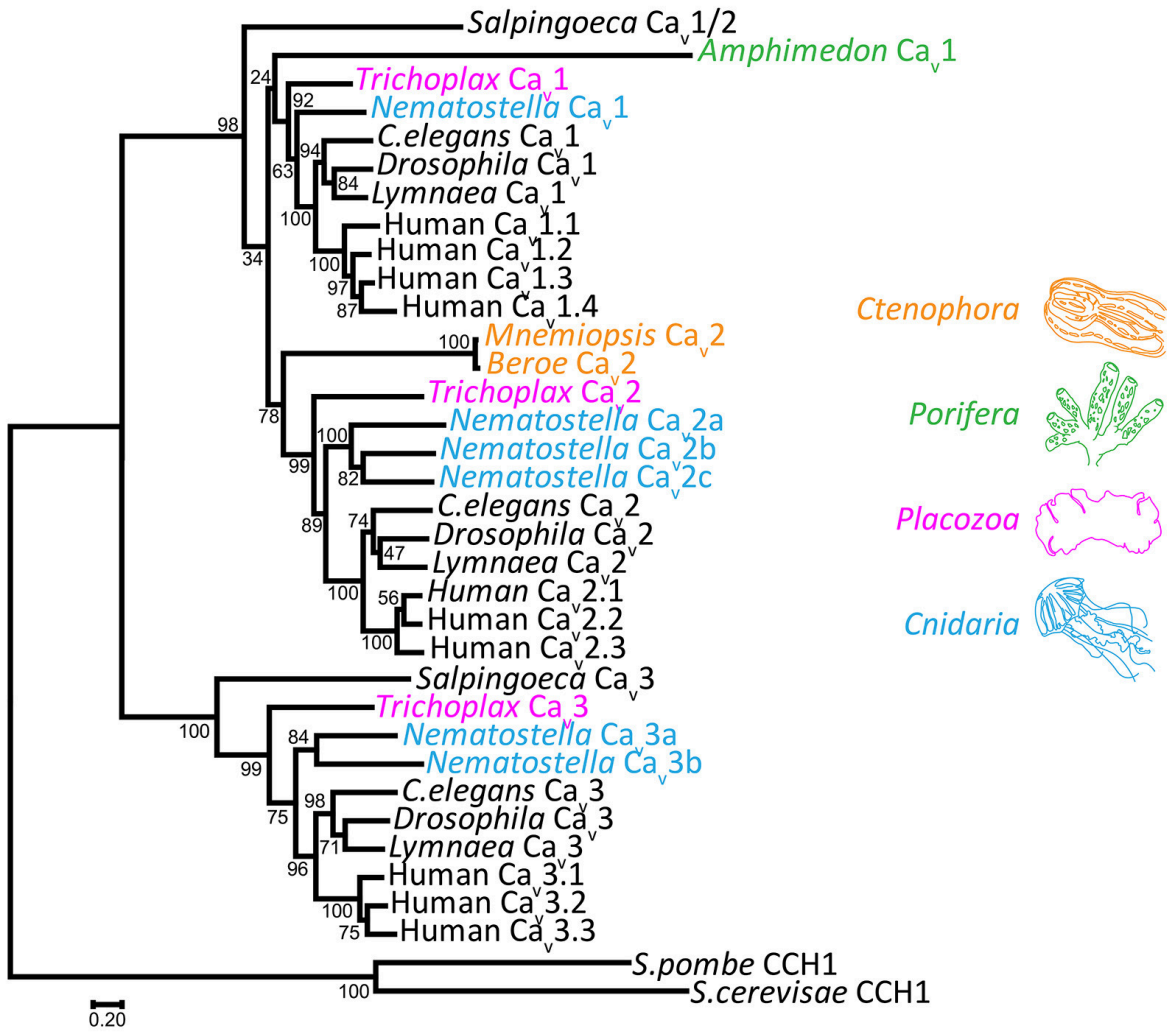
**Maximum likelihood protein phylogeny of select Ca_v_ channels from animals, rooted with Ca_v_ channel homologs from fungi**. Inference was achieved using MUSCLE protein alignment with MEGA7, followed by alignment trimming with TrimAL. Evolutionary models for maximum likelihood phylogenetic inference were tested with MEGA7, indicating that the LG matrix with gamma frequencies was the best fit using both corrected Akaike's Information Criterion and Bayesian Information Criterion. Node support values from 500 bootstrap replicates are indicated. GenBank accession numbers: *Salpingoeca* Ca_v_1/Ca_v_2: XP_004989719; *Amphimedon* Ca_v_1/Ca_v_2: XP_003383036; *Trichoplax* Ca_v_1: XP_002108930; *Trichoplax* Ca_v_2: XP_002109775; *Trichoplax* Ca_v_3: KJ466205; *C.elegans* Ca_v_1 (egl-19): NP_001023079; *C.elegans* Ca_v_2 (unc-2): NP_001123176; *C.elegans* Ca_v_3 (cca-1): CCD68017; *Drosophila* Ca_v_1 (α1-D): AAF53504; *Drosophila* Ca_v_2 (cacophony): AFH07350; *Drosophila* Ca_v_3 (Ca-α1T): ABW09342; *Lymnaea* Ca_v_1: AAO83839; *Lymnaea* Ca_v_2: AAO83841; *Lymnaea* Ca_v_3: AAO83843; human Ca_v_1.1: NP_000060.2; human Ca_v_1.2: AAI46847.1; human Ca_v_1.3: NP_001122312.1; human Ca_v_1.4: NP_005174.2; human Ca_v_2.1: O00555.2; human Ca_v_2.2: NP_000709; human Ca_v_2.3: NP_001192222.1; human Ca_v_3.1: NP_061496; human Ca_v_3.2: NP_066921; human Ca_v_3.3: NP_066919; *Mnemiopsis* Ca_v_2: AEF59085; *S.cerevisae* CCH1: P50077; *S.pombe* CCH1: NP_593894.1. Other accession numbers: *Nematostella* Ca_v_1: JGI-Genome Portal protein ID 88037; *Nematostella* Ca_v_2a, Ca_v_2b, Ca_v_2c, Ca_v_3a, Ca_v_3b: Transcript sequences from the sequenced transcriptome (Fredman et al., 2013) NVE4667, NVE18768, NVE1263, NVE5017, and NVE7616 respectively. Scale bar represents the number of amino acid substitutions per site along the sequence alignment.

Genomics has greatly improved our understanding of Ca_v_ channel molecular evolution. In vertebrates, gene duplications expanded the Ca_v_ channel repertoire to four Ca_v_1 channels (i.e., Ca_v_1.1 to Ca_v_1.4, collectively dubbed L-type channels), three Ca_v_2 channels (Ca_v_2.1 or P-/Q-type, Ca_v_2.2 or N-type, and Ca_v_2.3 or R-type channels) and three Ca_v_3 channels (Ca_v_3.1 to Ca_v_3.3 or T-type channels) (Perez-Reyes, 2003; Yu and Catterall, 2004; Jegla et al., 2009) (Figure 4). Ca_v_ channel genes independently expanded in cnidarians, such as the sea anemone *Nematostella vectensis*, to produce three Ca_v_2 channel genes (Ca_v_2a, Ca_v_2b, and Ca_v_2c), two Ca_v_3 channel genes (Ca_v_3a and Ca_v_3b), and a single Ca_v_1 channel gene (Moran and Zakon, 2014). Protostome invertebrates, such as arthropods, nematodes, and molluscs, as well as placozoans, all retain single genes for each of the three types of Ca_v_ channels (Figures 1, 3). Homologs of Ca_v_ channels and their subunits are present in the genomes and transcriptomes of pre-metazoans and early-diverging animals. However, extensive loss of ion channel gene content in these lineages (Liebeskind et al., 2015), combined with an unresolved phylogeny at the base of Metazoa, has made it difficult to define their evolutionary relationships with absolute certainty (Moran and Zakon, 2014). Ca_v_3 channels appear absent in ctenophore and sponge genomes, but are present in choanoflagellates (Fairclough et al., 2013), indicating that they predate animals but were likely lost in Ctenophora and Porifera (Moran and Zakon, 2014). Thus, *Trichoplax adhaerens* is the most basal extant animal known to possess *bona fide* homologs for all three types of cnidarian/bilaterian Ca_v_ channels types (Senatore et al., 2012) (i.e., Ca_v_1–Ca_v_3; Figure 4). Ca_v_1 and Ca_v_2 channels have more ambiguous phylogenies. In a recent study, the single Ca_v_ channel from sponge *A. queenslandica* was found to form a sister clade with Ca_v_1 and Ca_v_2 channels (hence dubbed Ca_v_1/2), as did a Ca_v_ channel from choanoflagellate *Salpingoeca rosetta* (Moran and Zakon, 2014). Thus, the authors proposed that Ca_v_1 and Ca_v_2 channels emerged via gene duplication of an ancestral Ca_v_1/2 channel, either early in Metazoa, or just before its emergence. Notably, node support for the phylogenetic position of the *Amphimedon* Ca_v_ channel was low, and in our hands, the channel clusters with Ca_v_1 channels under maximum likelihood inference, albeit with poor bootstrap support (Figure 4). Instead, the single Ca_v_ channels from ctenophores *M. leidyi* and *Beroe ovata* cluster with Ca_v_2 types (Moran and Zakon, 2014) (Figure 4). Clearly, the phylogeny of Ca_v_ channels at the base of Metazoa requires further analysis, perhaps resolvable via inclusion of additional Ca_v_ channel protein sequences from early and pre metazoans as they become available, and resolution of the phylogeny of Porifera vs. Ctenophora. With respect to protein sequence, the different Ca_v_ channels from the four basal metazoan phyla of Cnidaria, Placozoa, Porifera, and Ctenophora share canonical voltage sensors, appropriate selectivity filters of EEEE (i.e., Ca_v_1, Ca_v_2, and Ca_v_1/2 types) or EEDD (Ca_v_3 type), a gating brake (Ca_v_3 channels), and C-terminal IQ motifs (Ca_v_1, Ca_v_2, and Ca_v_1/2 channels) (Figure 3). Indeed, in light of recent advances in cryo-electron microscopy for rendering Ca_v_ channel secondary, tertiary and quaternary structures (Wu et al., 2015, 2016), an interesting prospect is to evaluate the structural homology between distant Ca_v_ channels, perhaps shedding additional light on their evolution.

As alluded to above, HVA (but not LVA) channels have a functional dependency on accessory Ca_v_β, as well as Ca_v_α_2_δ subunits (Curtis and Catterall, 1984; Catterall et al., 2005), which regulate channel membrane expression and biophysical properties (Figure 2A). Ca_v_β subunits influence the biophysical properties of Ca_v_1 and Ca_v_2 channels, and bind cytoplasmic AID helices to also increase membrane trafficking through inhibition of proteosomal degradation (Altier et al., 2011) and masking of an endoplasmic reticulum retention signal (Bichet et al., 2000). Ca_v_α_2_δ subunits interact with the extracellular surface of the channels, and have more minimal consequences for biophysical properties (Yasuda et al., 2004). Like Ca_v_β however, they increase channel membrane expression, and seem to play a role in targeting Ca_v_2 type channels to pre-synaptic terminals (Cantí et al., 2005; Hoppa et al., 2012). Interestingly, whereas the Ca_v_α_2_δ subunit was initially thought to be tethered to the extracellular surface of the membrane via a C-terminal transmembrane helix (Figure 2A), recent evidence suggests that instead the subunit is completely extracellular, held in place via a glycophosphatidylinositol anchor (Davies et al., 2010; Kadurin et al., 2012; Wu et al., 2016). Similar to the Ca_v_ channel subunits, the accessory subunit genes underwent independent gene duplication events in several animal lineages, including vertebrates which bear four Ca_v_β and four Ca_v_α_2_δ subunit genes (Buraei and Yang, 2010; Dolphin, 2013). The Ca_v_α_2_δ subunit appears absent in the genomes of early diverging sponges, ctenophores and single celled eukaryotes (Moran and Zakon, 2014), thus possibly being of eumetazoan origin, while the Ca_v_β subunit has a more ancient ancestry, present in genomes of choanoflagellates (Dawson et al., 2013; Moran and Zakon, 2014).

It is worth noting that some invertebrate Na_v_ channels, also of the four-domain P-loop family, are capable of conducting Ca^2+^-selective currents (Zhou et al., 2004; Zhang et al., 2011; Barzilai et al., 2012; Gosselin-Badaroudine et al., 2016). Two phylogenetically distinct types of Na_v_ channels have been identified in animals, Na_v_1 and Na_v_2. Na_v_2 type channels are the most ancient, having emerged in single-celled eukaryotes before the divergence of animals and fungi (Cai, 2012). Most metazoans possess Na_v_2 channel genes, however, vertebrates lost this type and only possess Na_v_1 channels. Na_v_1 channels perhaps evolved from an Na_v_2-type channel strictly in bilaterians, and therefore, outside of vertebrates and a few other clades, most bilaterian possess both Na_v_1 and Na_v_2 channels. With respect to cation selectivity, Na_v_2 channels conduct Ca^2+^-selective currents, bearing selectivity filter motifs of DEEA which resemble glutamate/aspartate rich Ca_v_ channel filters of EEEE and EEDD (Liebeskind et al., 2011; Barzilai et al., 2012). In cnidarians, one Na_v_2 channel gene, Na_v_2.5, evolved high Na^+^-selectivity via alteration of its selectivity filter motif to DKEA. Here, the positively-charged lysine (K) likely disrupts high affinity binding of Ca^2+^ (with contributions from other regions of the P-loops) (Barzilai et al., 2012), producing a Na^+^-selective pore. Instead, Na_v_1 channels independently evolved high Na^+^ selectivity, via a glutamate to lysine conversion in domain III of the selectivity filter (i.e., DEKA). Of the four most early-diverging metazoan phyla, none possess Na_v_1-type channels, cnidarians possess upwards of five Na_v_2 channels (e.g., *N. vectensis* has four Ca^2+^-selective DEEA channels, Na_v_2.1-Na_v_2.4, and one Na^+^-selective DKEA channel, Na_v_2.5). Ctenophores and *Trichoplax* each possess two Ca^2+^-selective DEEA channels (Na_v_2a and Na_v_2b), and sponges appear to have lost Na_v_2 channels (Liebeskind et al., 2011; Barzilai et al., 2012). Thus, an important caveat is that recorded voltage-gated Ca^2+^ currents in preparations where ion channel molecular identity is not known, could be attributed to Na_v_2 instead of Ca_v_ channels.

## Ca_v_ channel physiology in basal metazoans

### Cnidaria

Cnidaria is an ancient and diverse phylum with animals falling into two major clades, the Anthozoa (e.g., sea anemones, sea pens, and corals) and the Medusozoa (jellyfish and hydra) (Putnam et al., 2007). Common to all cnidarians is a relatively simple nervous system, organized as nets of synaptically connected neurons with minimal condensation into centralized neuronal structures (Katsuki and Greenspan, 2013). This organization is thought to resemble a primitive state, which is also found in ctenophores and contrasts the more centralized structures of bilaterians. The phylogenetic proximity of Cnidaria to Bilateria (Figure 1), and the absence of nervous systems in both placozoans and sponges, suggests that cnidarians and bilaterians share common ancestry for the nervous system. This is certainly apparent in the sequenced genomes and predicted proteomes of various cnidarians (Putnam et al., 2007; Chapman et al., 2010; Shinzato et al., 2011; Baumgarten et al., 2015), where they have more homologs of key bilaterian nervous system genes compared to more basal animals (Moroz and Kohn, 2015, 2016). Interestingly, cnidarian proteomes also have more PDZ protein-protein interaction motifs compared to pre-metazoans and more early-diverging animals, and less than bilaterian animals which possess more complex nervous systems. This is notable because PDZ motifs play important roles in synaptic protein scaffolding, and their expansion might have contributed to synapse evolution and complexification (Sakarya et al., 2010).

#### Pre-synaptic exocytosis

In the pre-synaptic terminal of bilaterians, Ca_v_2 channels play a dominant role in coupling excitation with fast pre-synaptic exocytosis. Ca_v_1 and/or Ca_v_3 channels provide more peripheral contributions, or contribute to other forms of excitation-secretion coupling such as neuroendocrine secretion (Ca_v_1 and Ca_v_3 channels) and low-threshold exocytosis (Ca_v_3 channels) (Carbone et al., 2006a; Simms and Zamponi, 2014). One requirement for fast synaptic transmission is the proximal coupling of pre-synaptic Ca_v_2 channels with Ca^2+^-sensitive proteins of the exocytotic machinery (e.g., synaptotagmin, complexin), such that transient “nanodomain” Ca^2+^ plumes, restricted to roughly 100 nanometer radii from the channel pore, effectively saturate the exocytotic apparatus (Clapham, 2007; Stanley, 2016). Two modes for functional coupling of Ca_v_2 channels with exocytotic proteins appear to exist: (1) In nanodomain coupling, direct, physical interaction of Ca_v_2 channels with proteinaceous elements of docked pre-synaptic vesicles allows single channels to trigger exocytosis of single vesicles (i.e., one-to-one coupling); (2) Instead, microdomain coupling involves a slightly more distal apposition between Ca_v_ channels and synaptic vesicles, without necessarily direct physical contact. Here, numerous Ca_v_ channel Ca^2+^ nanodomains sum into larger “microdomains,” which trigger exocytosis of numerous docked vesicles (i.e., group-to-group coupling) (Stanley, 2016).

Fast chemical synaptic transmission in cnidarians, as in bilaterians, requires Ca^2+^ influx through voltage-gated calcium channels (Bullock, 1943; Kerfoot et al., 1985). However, whether cnidarian Ca_v_2 channels similarly act as major drivers of pre-synaptic exocytosis remains to be determined. A recent study of the spatial expression of Ca_v_ channel mRNAs in the developing anthozoan sea anemone *N. vectensis* (Moran and Zakon, 2014), revealed that all of its Ca_v_ channel genes, including its three Ca_v_2 channels, are expressed in regions that overlap with expressed neurogenic marker genes ELAV and Musashi identified in a separate study (Marlow et al., 2009). However, direct evidence that Ca_v_2 channels are expressed in cnidarian neurons and exhibit pre-synaptic localization has yet to be provided. Indirectly, electrophysiological recordings of motor neurons from the hydrozoan jellyfish *Polyorchis penicillatus* reveal a prominent HVA Ca^2+^ current that resembles Ca_v_2 channels by lacking fast Ca^2+^/calmodulin-dependent inactivation (Przysiezniak and Spencer, 1992), a conserved feature of protostome and deuterostome Ca_v_1-type channels (Peterson et al., 1999; Spafford et al., 2006; Taiakina et al., 2013), also evident for the cloned and ectopically expressed Ca_v_1 channel from jellyfish *Cyanea capillata* (Jeziorski et al., 1998). Interestingly, neuromuscular junction (NMJ) synapses in *Polyorchis* have properties suggestive of nanodomain (one-to-one) coupling. *In vitro* voltage-clamp recording across the NMJ revealed that rapid and transient pre-synaptic Ca^2+^ influx elicits stronger post-synaptic responses than slower, more long-lasting Ca^2+^ influx, despite the former providing less total Ca^2+^ into the pre-synaptic terminal (Spencer et al., 1989). The increased efficiency for synaptic transmission with faster onset Ca^2+^ influx suggests that endogenous mechanisms for Ca^2+^ sequestration and extrusion impose spatial/temporal constraints on the calcium channel's ability to activate the exocytotic machinery. Instead, efficient transmission at this particular synapse requires fast plumes of cytoplasmic Ca^2+^, consistent with nanodomain coupling. Synapses with microdomain coupling tend to improve their efficacy (i.e., facilitate) with increased pre-synaptic Ca^2+^ influx, such as occurs during a burst of action potentials (Stanley, 2016). Such Ca^2+^-dependent facilitation has been observed in other cnidarian synapses (Roberts and Mackie, 1980), suggesting they are similar to vertebrates in having different synapses with either nanodomain or microdomain coupling, depending on developmental state or physiological requirements (Stanley, 2016).

Given the similar genomic content of pre-and post-synaptic genes between bilaterians and cnidarians, and their shared ancestry of the nervous system, it will be interesting to evaluate the homology in their mechanisms for synaptic transmission at the molecular level. With respect to nanodomain tethering of Ca_v_2 channels, there is at least evidence for homology between protostomes and deuterostomes, which diverged roughly 520 Mya (Blair and Hedges, 2005), around when medusozoans and anthozoans diverged from each other (Putnam et al., 2007). In both *Drosophila* and mouse, the presynaptic scaffolding protein Rab-3 interacting molecule (RIM) is essential for the appropriate pre-synaptic localization of Ca_v_2 channels, forming part of a molecular bridge between the channels and synaptic vesicles (Han et al., 2011; Kaeser et al., 2011; Graf et al., 2012). The interaction seems to occur via a RIM PDZ motif that binds the Ca_v_2 channel C-terminus; in mouse, targeted deletion of the RIM PDZ disrupts proper channel localization and synaptic transmission, and a direct physical interaction was observed between this motif and the channel C-terminus via yeast-two hybrid and NMR spectroscopy assays. However, a similar interaction was not observed in the chick synapse using co-immunoprecipitation (Khanna et al., 2006; Wong and Stanley, 2010), and a separate study found that RIM tethering of Ca_v_2 channels requires the Ca_v_β subunit to serve as an intermediary between the two proteins (Kiyonaka et al., 2007). Recently, evidence has emerged that Ca_v_2 channel pre-synaptic scaffolding undergoes a developmental switch in *Drosophila*, where different mRNA splice isoforms of the vesicle priming protein UNC-13 interact with distinct scaffolding proteins for either microdomain tethering in immature synapses (i.e., with Syd-1 and Liprin-α), or nanodomain tethering in mature synapses (i.e., with Bruchpilot and RIM-associated protein complexes) (Böhme et al., 2016). Thus, although it appears as though RIM plays conserved roles in Ca_v_2 channel tethering in protostome and deuterostome synapses, complex and dynamic processes are likely at play. In accordance, pre-synaptic scaffolding proteins Mint1 and CASK, have been also been found to contribute to proximal coupling of Ca_v_2 channels with the exocytotic machinery in both rodents (Maximov and Bezprozvanny, 2002) and the mollusc snail *Lymnaea stagnalis* (Spafford et al., 2003). Furthermore, unique specializations appear in distinct lineages, such as vertebrate synaptic protein interaction (“synprint”) sites in the II-III linkers of vertebrate Ca_v_2.1 and Ca_v_2.2 channels, which directly interact with vesicular SNARE complex proteins syntaxin-1A/B and SNAP-25 to regulate channel pre-synaptic tethering and gating (Sheng et al., 1994; Rettig et al., 1996).

As noted above, low voltage activated Ca_v_3 type channels are implicated in “low threshold exocytosis,” occurring in neuroendocrine cells (Carbone et al., 2006a,b) and neurons capable of graded synaptic transmission (Weiss et al., 2012; Weiss and Zamponi, 2013). Though less well documented than spike-dependent transmission (i.e., elicited by action potentials and Ca_v_2 channels), graded transmission plays important roles in certain neurophysiological contexts in both vertebrates and invertebrates. In invertebrates, LVA calcium channels and graded synaptic transmission play major roles in the activity of intrinsically rhythmic neural circuits (i.e., central pattern generators or CPGs), such as the interneuron network that drives heart contraction in the protostome leech (Angstadt and Calabrese, 1991; Lu et al., 1997). In vertebrates, Ca_v_3 channels also contribute to graded synaptic transmission, including in the retina, and between neurons located in the central and peripheral nervous systems (Weiss and Zamponi, 2013). The first detailed description of graded vs. spike-dependent transmission came from leech interneurons of the heart CPG, where classical spike-dependent synaptic transmission, driven by an HVA Ca_v_ channel (perhaps Ca_v_2), was found to co-exist in the same neurons with graded transmission driven by an LVA Ca_v_ channel (perhaps Ca_v_3) (Angstadt and Calabrese, 1991; Lu et al., 1997). Here, two alternate modes of cellular excitability, action potentials vs. sub-threshold plateau potentials, were respectively found to elicit strong or graded inhibitory post-synaptic responses between paired CPG neurons. Subsequently, similar bimodal cellular excitability and synaptic transmission was documented in the vertebrate olfactory bulb (Egger et al., 2003).

Interestingly, “bimodal” excitability and graded vs. spike-dependent synaptic transmission have also been documented in the neuromuscular junction of medusozoan jellyfish *Aglantha digitale*. Here, neuromuscular synapses of large axon motor neurons manifest either low-threshold, spike-independent synaptic transmission, which elicit graded contractions of the bell myoepithelium during slow pelagic swimming, or spike-dependent synaptic transmission, triggered by aggressive, predatory tactile cues, which elicit strong contractions of the bell during fast escape swimming (Mackie, 1980; Roberts and Mackie, 1980; Kerfoot et al., 1985; Mackie and Meech, 1985; Meech and Mackie, 1993). During pelagic swimming, spontaneous depolarizing synaptic inputs into motor neurons activate an LVA channel resembling a Ca_v_3 type, generating low threshold Ca^2+^ spikes with peak depolarization just below action potential threshold (i.e., about −25 millivolts or mV). These subthreshold Ca^2+^ spikes occur as spontaneous bursts at a rate of about 3–4 per second (Meech and Mackie, 1993), and trigger exocytosis and mild graded contractions in myoepithelial striated muscle cells. In the same axons, strong depolarizing sensory inputs generate Na^+^-dependent action potentials, which trigger all-or-none exocytosis and transmission producing much stronger contraction of the bell myoepithelium for the escape response. Of note, the data does not rule out the possibility that the *Aglantha* Ca_v_3-like channel activates an HVA Ca_v_ channel which in turn associates with the exocytotic machinery. However, the peak of the LVA Ca^2+^ spike only reaches about −25 mV (Meech and Mackie, 1993), which is barely at the activation threshold for recorded HVA Ca_v_ channels from cnidarians (Przysiezniak and Spencer, 1992; Jeziorski et al., 1998). Thus, the LVA calcium channel observed in *Aglantha* axons might well be positioned within nanometer proximity of the exocytotic machinery, able to directly activate exocytosis. Such an association is not without precedent: The three vertebrate Ca_v_3 channel isotypes were recently found to directly interact with core SNARE proteins syntaxin-1A (all three channel isotypes) and SNAP-25 (only Ca_v_3.2), and disruption of the syntaxin-Ca_v_3.2 channel interaction was found to abrogate the channel's contribution to low-threshold exocytosis in a neuroendocrine cell line (Weiss et al., 2012).

#### Muscle contraction

Whereas both Ca_v_1 and Ca_v_2 type channels are expressed in bilaterian neurons and neuroendocrine cells, Ca_v_1 channels are often the only type found in smooth, cardiac, and striated muscle, with a few instances of Ca_v_3 channel expression (Ren et al., 1998; Jeziorski et al., 2000; Jospin et al., 2002; Catterall, 2011; Senatore et al., 2014). Thus, in most smooth and cardiac muscle cells, Ca^2+^ influx through L-type/Ca_v_1 channels serves to directly activate contractile proteins, and to trigger further increases in cytoplasmic Ca^2+^ by activating ryanodine receptors in the sarco/endoplasmic reticulum (SER) (i.e., calcium-induced calcium release or CICR)(Reuter, 1979; Tsien, 1983; Bers, 2002). In vertebrate skeletal muscle, Ca_v_1 channels have evolved a specialized ability to sidestep the CICR process. Here, membrane-localized Ca_v_1 channels directly interact with SER ryanodine receptors; activation of Ca_v_1 channels at the membrane relays conformational changes in the ryanodine receptor leading to release of SER Ca^2+^, without a need for Ca_v_1 channel Ca^2+^ influx (Tanabe et al., 1990, 1993; Catterall, 1991).

Interestingly, the coupling of cytoplasmic Ca^2+^ influx with rapid activation of contractile proteins seems to be a metazoan innovation. A recent in-depth genomic study found that the Ca^2+^- calmodulin (CaM)—myosin light chain kinase (MLCK) cascade, critical for excitation-contraction coupling, occurs strictly in metazoans where MLCK is absent in the genomes of choanoflagellates and other non-metazoan organisms (Steinmetz et al., 2012). Also interesting is that although “core” contractile proteins appear to have been present prior to the emergence of Metazoa, key proteins associated specifically with striated muscle in bilaterians are absent in cnidarians and ctenophores, which also possess striated muscle. As such, fast-twitching striated muscle likely evolved independently between at least bilaterians and cnidarians/ctenophores (Burton, 2008; Steinmetz et al., 2012).

Outside of a few species of swimming sea anemones, anthozoans are mostly devoid of striated muscle for contractile movement, bearing primitive smooth muscle cells with roles in feeding (mouth and tentacles) and digestion/reproduction (gastrovascular cavity) (Chapman, 1974; Burton, 2008). Medusozoans, which can become motile medusae (i.e., jellyfish), possess extensive striated musculature (“muscle sheets”) in the bell epithelium for swimming, in addition to smooth muscle cells (Chapman, 1974; Burton, 2008). The striated swimming muscles seem to lack extensive SER structures (Chapman, 1974; Keough and Summers, 1976; Singla, 1978; Spencer, 1979), suggesting a reduced dependency on CICR in lieu of Ca^2+^ influx through plasma membrane Ca^2+^ channels. Accordingly, removal of external Ca^2+^ during intracellular recording of bell striated muscle cells from *Aglantha* completely abrogates muscle action potentials and contraction (Kerfoot et al., 1985). Notably, the action potentials of these muscle cells are slow to reach peak (Kerfoot et al., 1985) compared to the Na^+^-dependent action potentials of their pre-synaptic effector neurons (Mackie and Meech, 1985; Meech and Mackie, 1993). This suggests the absence of fast, Na^+^-selective Na_v_ channels for depolarization (i.e., Na_v_2.5-like channels, Barzilai et al., 2012). Instead, the long-lasting and complex waveform of *Aglantha* muscle action potentials (Roberts and Mackie, 1980) indicates that multiple Ca^2+^ conductances are at play, perhaps for endowing the cells with a capacity to respond to bimodal pre-synaptic innervation as discussed above (Kerfoot et al., 1985).

Long-lasting action potentials have also been observed in striated swimming muscle of another hydrozoan medusa, *P. penicillatus*. Here, intracellular recordings revealed action potentials whose depolarization depends on both Na^+^ and Ca^2+^ influx (Spencer and Satterlie, 1981). Like in *Aglantha* swim muscles, these spikes also exhibits long lasting plateau phases, reminiscent of vertebrate cardiac muscle in which prolonged Ca^2+^ influx through Ca_v_1 channels ensures effective contraction of the heart for expulsion of blood (Grant, 2009). Given the similarity in waveforms between vertebrate cardiac muscle and jellyfish swimming muscle, and the predominance of Ca_v_1 channels in driving bilaterian muscle contraction, it is tempting to speculate that the single Ca_v_1 channel in cnidarians also drives muscle contraction. However, the data is sparse in this regard and other types of Ca^2+^ permeable channels could certainly be involved. As noted earlier, the most detailed molecular description of cnidarian Ca_v_1 channels comes from the cloning and ectopic expression of a homolog from schyphozoan jellyfish *C. capillata* (Jeziorski et al., 1998). When expressed in *Xenopus* oocytes, *Cyanea* Ca_v_1 behaves like protostome and deuterostome Ca_v_1 channels by exhibiting high voltage of activation/inactivation, as well as apparent Ca^2+^-dependent inactivation evidenced by more rapid decay of its macroscopic currents in the presence of external Ca^2+^ compared to Ba^2+^.

#### Unique Ca_v_ channel physiology

Cnidarians get their name from cnidocytes, or “stinging cells,” best known for their role in jellyfish tentacles where they discharge thread-like tubules laced with painful and sometimes lethal toxins for defense and predation. As cnidocytes can only be used once, their discharge is highly regulated, especially those involved in prey capture (Anderson and Bouchard, 2009). Regulation involves a convergence of chemosensory and mechanosensory neural synaptic inputs (Pantin, 1942; Westfall, 2004; Anderson and Bouchard, 2009), intrinsic mechano- and chemo-sensitivity of the cnidocytes themselves (Brinkmann et al., 1996; Thurm et al., 1998, 2004), and cnidocyte-cnidocyte communication either directly via gap junctions (Mire et al., 2000; Price and Anderson, 2006), or through local synaptic circuits between cnidocytes and intermediate sensory cells located nearby (Holtmann and Thurm, 2001a). Notably, only hydrozoans and perhaps anthozoan sea anemones bear gap junction genes (Putnam et al., 2007; Chapman et al., 2010; Shinzato et al., 2011; Baumgarten et al., 2015), and electrical coupling between cnidocytes need not occur, even when gap junction genes are likely present (Holtmann and Thurm, 2001a,b).

Intracellular recording has revealed that cnidocytes are highly electrically active. Application of species-specific prey extracts via perfusion in sea water saline evokes depolarizing synaptic potentials and bursts of action potentials in impaled tentacle cnidocytes (Brinkmann et al., 1996; Price and Anderson, 2006); perfusion of Ca^2+^-free saline and calcium channel blocker Ni^2+^ disrupt this induced activity, likely through pre-synaptic disruption Ca_v_ channels and exocytosis (Price and Anderson, 2006). Interestingly the cnidocyst, an endomembrane-derived organelle harboring the cnidocyte stinging thread, is thought to resemble synaptic and neuroendocrine vesicles in that its exocytotic discharge depends on both membrane depolarization and Ca^2+^ influx (Skaer, 1973; Gitter et al., 1994). Patch clamp recording of cnidocytes has failed to directly identify Ca_v_ channel currents, but this has been attributed to washing out of endogenous currents during patch clamp recording (Anderson and Bouchard, 2009). Furthermore, *in situ* localization of Ca_v_ channel transcripts in *N. vectensis* revealed that one of the three Ca_v_2 channel isotypes, Ca_v_2a, is strongly expressed in cnidocytes (Moran and Zakon, 2014), and both a full length Ca_v_β subunit and a fragment of an unspecified Ca_v_ channel have been detected in cnidocyte-specific mRNA from the Portugese man'o war (*Physalia physalis*) (Bouchard et al., 2006; Dunn, 2009). Thus, it is likely that *in vivo* Ca_v_ channels contribute to exocytosis of the cnidocyst, perhaps using similar machinery used for pre-synaptic and neuroendocrine secretion. However, cnidocyst exocytosis likely involves additional molecular adaptations that prevent exocytosis in the absence of proper chemosensory and mechanosensory inputs (Anderson and Bouchard, 2009).

Finally, Ca_v_ channels have been implicated in calcification of corals, which accumulate CaCl_2_ at a rate of about 10 kg per meter squared of coral reef per year (Chave et al., 1972). Application of phenylalkylamine and dihydropyridine Ca_v_1 channel blockers attenuates calcification in corals *Stylophora pistillata* (Tanbutté et al., 1996) and *Galaxea fascicularis* (Marshall, 1996), and a cloned Ca_v_1 channel gene was detected as an expressed protein in the calicoblastic ectoderm of *Stylophora*, which is involved in calcium carbonate precipitation (Zoccola et al., 1999). Thus, for at least some species of coral, Ca_v_ channels might contribute to the calcification process, but certainly other Ca^2+^-handling channels, pumps and exchangers also likely contribute (Marshall, 1996; Tanbutté et al., 1996).

### Placozoa

**Figure 5:**
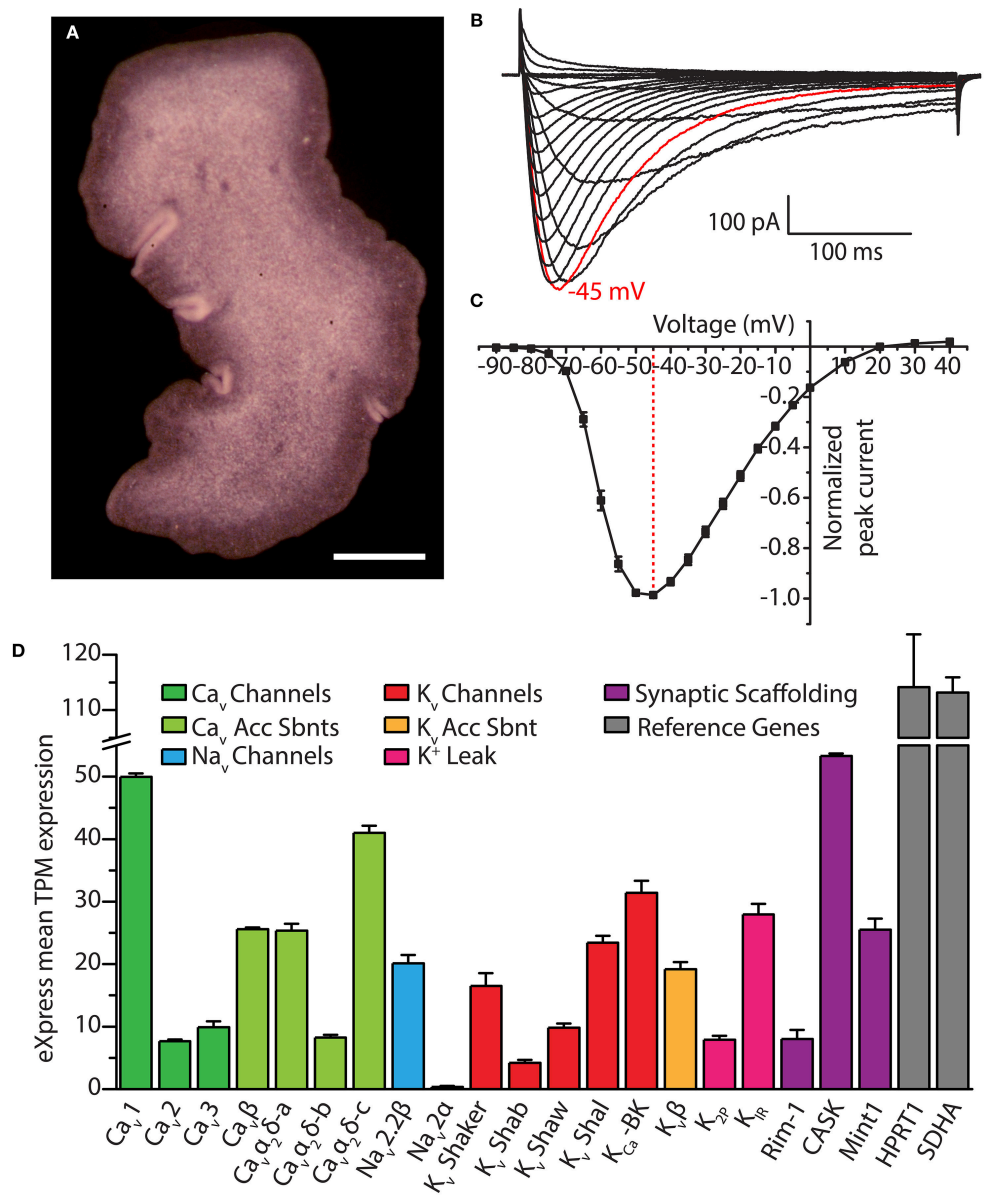
**(A)** Dorsal view of *Trichoplax adhaerens* photographed through a stereomicroscope, revealing its irregularly-shaped body lacking symmetry outside of dorsal-ventral polarity. Gland cells are located in the ventral epithelium, most concentrated along the outside rim (i.e., within the darker band visible in the image). Scale bar is 200 μm. **(B)** Whole cell patch-clamp recorded Ca^2+^ currents of the cloned *Trichoplax* Ca_v_3 channel expressed in HEK-293T cells, bearing rapid activation and inactivation kinetics, and a crossing over of current traces during inactivation with increasing depolarization (recorded in 2 mM external Ca^2+^ solution). **(C)** Current-voltage plot of average normalized peak inward Ca^2+^ current of *Trichoplax* Ca_v_3, revealing its low voltage of activation with peak inward current at −45 mV (*n* = 10, error bars indicate SE from the mean). **(D)** Bar graph of mean mRNA expression levels of select *Trichoplax* ion channel genes and their subunits estimated with the program eXpress (Roberts and Pachter, 2013), quantified as transcripts per million (TPM) from assembled transcriptome data and four separate Illumina sequencing datasets of whole animal poly(A)-extracted mRNA (2x125 base pair reads; manuscript in preparation). Channels were identified via BLAST homology with mammalian protein sequences using an expect value cut-off of 1 x 10^−5^. Two ubiquitously expressed genes, Hypoxanthine Phosphoribosyltransferase 1 (HPRT1) and Succinate Dehydrogenase A (SDHA), are included as reference genes. Error bars indicate standard error from the mean TPM expression.

Although the genome was sequenced for *T. adhaerens* (Srivastava et al., 2008), the only identified species of the phylum Placozoa, we understand little about placozoan species diversity, life cycle, reproduction or ecology (Schierwater, 2005; Eitel et al., 2013). *Trichoplax* was first discovered in 1883 by German zoologist F. E. Schulze, residing in a seawater aquarium in Austria (Schulze, 1883). Schulze named this peculiar animal based on its flat, hairy (*tricho*) plate (*plax*)-like appearance (Figure 5A), attributed to its ciliated epithelium used for adhering (*adhaerens*) to and gliding along hard surfaces. After its initial discovery and a few years thereafter, *Trichoplax* was largely forgotten until being rediscovered nearly a century later in the 1960s, spurring a new wave of research. Recently, interest has piqued again in light of the available genome sequence, and the phylogenetic placement of *Trichoplax* as sister to cnidaria/bilateria (Figure 1). This position makes *Trichoplax* a relevant subject for studying the evolution of complex animal traits such as development, body patterning and nervous system function, since it lacks these features yet harbors most genes necessary for their implementation and function (Srivastava et al., 2008). *Trichoplax* is a small marine invertebrate (i.e., 0.1–1 mm diameter; Figure 5A), which lives in shallow tropical and subtropical ocean waters, and can be easily grown in the lab where it divides asexually via binary fission, needing only a supply of healthy living algae for nourishment (Heyland et al., 2014; Smith et al., 2015). Microscopy studies indicate that *Trichoplax* possesses only six cell types, the least of any known animal, with no evidence for either chemical or electrical synapses, nor organized muscle fibers (Grell and Benwitz, 1971, 1974; Rassat and Ruthmann, 1979; Smith et al., 2014). Remarkably, despite these absences, *Trichoplax* is able to carry out motile behavior including feeding, chemotaxis and phototaxis (Ueda et al., 1999; Heyland et al., 2014; Smith et al., 2015). Unfortunately, electrophysiological recording of *Trichoplax* cells has yet to be reported, so we know little about the roles of ion channels and electrical excitability in *Trichoplax* biology. However, the persistence of electrogenic genes in its genome, including single representatives for each type of Ca_v_ channel (Ca_v_1-Ca_v_3; Figure 4), suggests that electrical and Ca^2+^-signaling do occur in *Trichoplax*. Here, we include in the discussion some of our own ongoing research, characterizing *Trichoplax* Ca_v_ channels.

#### Neurosecretory-like gland cells

Of the six documented *Trichoplax* cell types, gland cells most resemble neurons and neurosecretory cells in their expression of exocytotic SNARE proteins and membrane-apposed vesicles (Syed and Schierwater, 2002; Smith et al., 2014). Gland cells are concentrated around the periphery of the flat irregularly-shaped animal (Figure 5A), with some sparsely distributed along the ciliated ventral epithelium. Interestingly, gland cell vesicles exhibit cell-specific variability in electron density when observed under transmission electron microscopy (Smith et al., 2014). Thus, some gland cells appear specialized for secreting regulatory peptides, many of which are predicted from the genome (Nikitin, 2015) (i.e., those bearing electron dense vesicles), while others perhaps transmit small molecules such as amino acids and biogenic amines (Srivastava et al., 2008) (pale vesicles). Heterogeneity of gland cell vesicular content is also apparent through immunolabeling with antibodies against RFamide and FMRFamide neuropeptides (Schuchert, 1993; Smith et al., 2014), which selectively label a subset of gland cells located on the animal's periphery. Indeed, key questions remain about the role of gland cells in *Trichoplax* physiology and behavior, however their apparent exocytotic nature, coupled with their peripheral localization, suggests that they exert paracrine control over other cells for coordinating activity (Smith et al., 2014, 2015). For example, *Trichoplax* feeding behavior begins with the animal pausing over patches of algae detected under its body, via coordinated cessation of beating monociliated cells on the ventral epithelium (Ueda et al., 1999; Smith et al., 2014). The detected algae are then lysed by hydrolytic secretions from lipophil cells, but only those positioned close by. The animal remains sessile after algal lysis, anchored along the periphery while internally located cells undergo a churning movement. Afterwards, ventral epithelial cells resume beating, and the animal glides on to new algae (Smith et al., 2015). Here, peripherally-located gland cells are optimally positioned to exocytose paracrine factors over the entire ventral epithelium (Smith et al., 2014, 2015). Instead, more centrally located gland cells are proposed to function as chemosensory cells, which exocytose factors to inform adjacent lipophil cells of the presence of algae (Singla and Reiter, 2006; Smith et al., 2015). Further work needs to be done to understand how *Trichoplax* cells are coordinated in the absence of synapses during feeding and other behaviors. However, the expression of SNARE proteins in gland cells, combined with the presence of Ca^2+^-sensitive elements of the exocytotic machinery in the genome (Srivastava et al., 2008) (e.g., synaptotagmin, synaptophysin, and complexin), hints that these particular cells exhibit Ca^2+^-dependent exocytosis.

Our ongoing research on *Trichoplax* Ca_v_ channels provides indirect support for the potential dependence on calcium for exocytosis in gland cells. We recently cloned and *in vitro* expressed the single T-type Ca^2+^ channel homolog from *Trichoplax*, finding that despite more than 600 million years of divergence from vertebrate channels, it bears the distinguishing structural features of this channel type including a selectivity filter motif of EEDD, and a predicted helix-loop-helix gating brake structure in the domain I-II linker (Figure 2B). Also conserved with other T-type channels are its biophysical properties, where *in vitro* recorded Ca^2+^ currents exhibit hallmark attributes including a low voltage of activation, rapid activation and inactivation kinetics (Figures 5B,C), and a reduced selectivity for Ca^2+^ over Na^+^ compared to HVA Ca_v_1 and Ca_v_2 channels (in review). Thus, it seems as though the basic structural and functional features of Ca_v_3 channels, optimized for regulating excitability and driving low threshold exocytosis, were established very early on during evolution and perhaps extend beyond *Trichoplax* to the even more primitive homolog identified in the genome of choanoflagellate *Salpingoecca rosetta* (Fairclough et al., 2013; Moran and Zakon, 2014).

Interestingly, immunoabeling with specific custom antibodies against TCa_v_3 produced labeling exclusively in gland cells, with intense staining along the outside edges where vesicular exocytosis is likely to take place (Smith et al., 2014). We also recently cloned and *in vitro* expressed the *Trichoplax* Ca_v_2 channel homolog, and, similarly, immunolabeling points to exclusive expression in gland cells, along with the HVA subunit Ca_v_α_2_δ (unpublished data). Thus, beyond the presence of exocytotic machinery and “neurosecretory” ultrastructural markers, gland cells resemble select neurons and neurosecretory cells in their expression of both HVA (Ca_v_2) and LVA (Ca_v_3) channels (Weiss and Zamponi, 2013). It will certainly be interesting to determine whether gland cells exhibit homologous interactions between Ca_v_2 and Ca_v_3 channels and proteins which in neurons and neurosecretory cells complex the channels within nanometers of the exocytotic machinery, as discussed above (e.g., RIM, Mint1 and CASK, and the SNARE proteins). We note from an ongoing transcriptomic study that mRNAs of numerous pre- and post-synaptic scaffolding genes are indeed expressed in *Trichoplax*, including RIM, Mint1 and CASK (Figure 5B). Also of interest will be to determine how gland cells and other cell types might be electrically activated in the absence of synaptic inputs, perhaps via paracrine signaling, or, via cell-intrinsic sensory pathways as occurs in cnidarian cnidocytes. Indeed, the nature and purpose of electrical signaling in *Trichoplax* remains a mystery. However, it is likely of significant importance, where in addition to Ca_v_ channels, the animal expresses mRNAs of a core set of genes required for generating action potentials and propagating fast electrical signals: One of the two Na_v_2 channels predicted from the genome (Srivastava et al., 2008; Liebeskind et al., 2011), K_v_ channels of the Shaker, Shab, Shal, and Shaw varieties, a Ca^2+^-activated K^+^ channel (i.e., large conductance BK), a K_v_ channel accessory β subunit, and 2-pore K^+^ (K_2P_) leak channels and inward rectifying K^+^ (K_IR_) channels, essential for setting the polarized resting membrane potential of excitable cells (Figure 5D).

#### Cellular contractility

It is interesting that of the three Ca_v_ channel types, the Ca_v_1 channel appears to be the most highly expressed in the *Trichoplax* transcriptome (Figure 5D), considering the specialized role that Ca_v_1 channels play in excitation-contraction coupling in muscle, and the absence of ultrastructural markers for muscle in *Trichoplax* (Smith et al., 2014). The animal possesses the core genetic elements required for the establishment and operation of rapidly contracting muscle cells, and in fact shares slightly more of these genes with bilaterians/cnidarians than do ctenophores (Steinmetz et al., 2012), which appear to have independently evolved muscle (Ryan et al., 2013). Still debated is whether *Trichoplax* represents a simplified animal (Ryan and Chiodin, 2015), where the absence of clear ultrastructural markers for muscle might reflect a lost or diminished phenotype. However, the retention of contractile genes in the genome indicates utility, where they might play roles in contractile cellular processes nonetheless, or perhaps serve completely different functions. In support of the former, *Trichoplax* performs movements that appear independent of the ciliated ventral epithelium, such as the churning motion during feeding, and folding or rippling along its edges (Heyland et al., 2014; Smith et al., 2015). It might be the case that rudimentary contractile mechanisms underlie these movements; fiber cells, which lie between the dorsal and ventral epithelia and have branched protrusions that contact all other cell types, have been proposed to mediate contractile movements (Schierwater, 2005). However, whether these or any other *Trichoplax* cell types employ contractile genes in a manner homologous to muscle remains to be determined, as is the role for the single Ca_v_1 channel.

#### Ciliary beating

*Trichoplax* ciliary locomotion presents distinct modalities, including starting and stopping, as well as rotation and direction changes (Ueda et al., 1999; Heyland et al., 2014; Smith et al., 2015). Although poorly understood, transitions in *Trichoplax* locomotive states are dependent on food concentration (Ueda et al., 1999), and might require altered beating modes of cilia that project from the ventral epithelium. In other eukaryotic cells, alterations in ciliary waveform depend on Ca_v_ channels (Quarmby, 2009). Well documented examples of alternate modes for ciliary beating come from ctenophores (discussed below), as well as single-celled protists such as paramecia and the green algae *Chlamydomonas reinhardtii*, which alter ciliary waveforms in response to external stimuli, temporarily changing swimming trajectory. Early studies on paramecia revealed that their ability to reverse upon mechanical stimulation relies on external Ca^2+^, whose transient influx specifically into cilia triggers a switch in the beat cycle (Naitoh, 1968; Kung and Naitoh, 1973). Ciliary beating in paramecia is referred to as the “ciliary” waveform, which consists of an asymmetric power stroke in one direction followed by a weaker recovery stroke in the other. The ciliary waveform is common in metazoans, exemplified in the human lung where epithelial cilia use this pattern to expel particles and fluid into the pharynx (Satir and Christensen, 2007). In paramecia, electrophysiological recording revealed that the calcium channels responsible for switching the direction of the ciliary power and recovery strokes, and hence direction of movement, reside exclusively along the cilia and not the cell body (Dunlap, 1977). *Chlamydomonas* also exhibits direction changes, where light or mechanical stimulation causes a switch in ciliary waveform. Normally, the two cilia of *Chlamydomonas* exhibit ciliary waveforms with the power strokes that pull the cell body forward; upon optical/mechanical stimulation, and in a Ca^2+^-dependent manner, the cilia switch to a symmetrical “flagellar” waveform (Bessen et al., 1980), much like that of swimming sperm, temporarily reversing movement such that cell body leads while the cilia push from behind. Here, light or mechanical stimulation generates a depolarizing membrane potential (Harz and Hegemann, 1991), activating a pre-metazoan Ca_v_ channel homolog positioned along the distal portion of the cilia (Fujiu et al., 2009), consistent with the calcium channel localization in paramecia cilia. Gene disruption of the *Chlamydomonas* channel, dubbed CAV2, abrogates both the light and mechanical induced reversal, indicating a convergence of the two sensory modalities on CAV2 channel activation (Matsuda et al., 1998; Fujiu et al., 2009).

The mechanisms by which Ca^2+^ influx controls ciliary waveform transition involves dynamic regulation of dynein motor complexes positioned between pairs of ciliary microtubules (Yang et al., 2001; Hayashi et al., 2002; Patel-King et al., 2002; Wargo and Smith, 2003, 2007). Notably, Ca_v_ channels and Ca^2+^ influx are not necessary for maintaining ciliary beating *per se*, and might be specific for altering waveforms under transient, induced conditions (Tamm, 1994, 2014a). A recent study looking at the roles of Ca_v_ channels in ciliary beating of mammalian ependymal cells, which move cerebral spinal fluid in the central nervous system, found no effect of Ca^2+^ influx on ciliary beating and fluid movement, where Ca_v_1 channels were found localized mostly in the cell soma (Doerner et al., 2015). Instead, Ca^2+^ influx through CatSper channels in sperm triggers hyperactivation of the flagellar beat (Qi et al., 2007). Indeed, whether the cilia on *Trichoplax's* dorsal and ventral epithelium exhibit alternate or modulated beating modes remains to be determined; if so, it will be interesting to evaluate whether Ca_v_ channels are involved.

### Porifera

Sponges are phylogenetically more basal than Placozoans (Figure 1), however, they are considerably more complex bearing at least 16 different cell types (Simpson, 2012) organized into various simplified tissues (Leys, 2015). Like *Trichoplax*, sponges lack synaptically-connected neurons and true muscle cells. However, there are at least two cell types, pinacocytes and actinocytes, which are thought to contract and bear some structural resemblance to muscle (Leys and Meech, 2006; Nickel et al., 2011). Sponges are sedentary and consume microorganisms such as bacteria and protozoans by filtering them from sea water, drawn through internal canals by the beating of ciliated choanocyte cells. The most obvious behaviors of sponges revolve around feeding. Glass sponges, named so because of their rigid silica skeletons, respond to excessive particulates in the water by propagating Ca^2+^-dependent electrical impulses along cellular syncytia in order to pause choanocyte ciliary beating and arrest the feeding current (Leys and Mackie, 1997; Leys and Meech, 2006). Most other sponges are soft-bodied and lack syncytia (i.e., are “cellular”), and instead contract their entry/exit points for water flow (i.e., ostia and osculum, respectively), or their entire aquiferous systems, in order to prevent particulates from getting into canals (Nickel, 2010; Nickel et al., 2011), or to expel them (Elliott and Leys, 2007). Another fairly well characterized sponge behavior is larval swimming, which serves for dispersal and location of suitable sea floor settling grounds (Maldonado and Bergquist, 2002). Swimming is mediated by beating cilia on the larval epithelium, sometimes arranged asymmetrically between the poles of oblong species (Maldonado and Bergquist, 2002). Interestingly, numerous sponge larvae exhibit phototactic swimming, mediated by rapid, light-induced changes in ciliary beating (Leys and Degnan, 2001; Leys et al., 2002; Leys, 2015), reminiscent of light-dependent ciliary responses in *Chlamydomonas* but mediated by different photosensitive effectors [i.e., channel rhodopsins in algae (Nagel et al., 2003) vs. cryptochromes in sponges (Leys et al., 2002; Rivera et al., 2012)].

As contenders for the most basal surviving animal phylum, poriferans are positioned to provide important insights into animal evolution. Recent genomic and transcriptomic studies reveal that sponges possess and express key genes associated with nervous system development and function (Srivastava et al., 2010; Conaco et al., 2012; Riesgo et al., 2014; Fernandez-Valverde et al., 2015; Guzman and Conaco, 2016). Understanding how these genes operate and interact in sponges *in vivo* can shed light on conserved and ancient modules of gene function which served as building blocks for nervous system evolution (Ryan and Grant, 2009). Several such insights have already emerged, such as the apparent co-expression of post-synaptic scaffolding genes in certain sponge tissues, most bearing conserved protein-protein interaction motifs required for synaptic complexing (Sakarya et al., 2007); the presence of subsets of genes involved in neurotransmitter biosynthesis and transport, as well as corresponding ionotropic and/or metabotropic receptors including those for GABA and L-glutamate (Srivastava et al., 2010; Riesgo et al., 2014; Moroz and Kohn, 2015); and in conjunction, physiological sensitivity to some of these transmitters, most evident by alterations in contractile behavior (Elliott and Leys, 2010; Leys, 2015). Interestingly however, outside of glass sponges Porifera appear mostly devoid of fast electrical impulses, and they lack both Na_v_ and K_v_ channels which mediate most action potentials (Tompkins-Macdonald et al., 2009; Srivastava et al., 2010; Riesgo et al., 2014), as well as gap junction genes which permit electrical coupling between cells (Leys, 2015). Thus, with respect to fast electrical signaling, sponges are likely simplified from the root metazoan ancestor, since most electrogenic genes are present in pre-metazoan genomes (Moran et al., 2015). We know little about the biological roles of the remaining electrogenic genes in sponges, including the single Ca_v_ channel whose phylogenetic relationship to other metazoan Ca_v_ channels remains unclear (Figure 4). In this section, we briefly highlight some of the few examples of poriferan physiology and behavior where Ca_v_ channels might possibly play a role, and further, discuss the atypical contraction of sponge cells where cell excitation and Ca_v_ channels appear not to be involved.

#### Cellular contractility

Placozoans and cellular sponges both exhibit quasi-coordinated contractile behavior in the complete absence of neurons and muscle. However, whereas *Trichoplax* has the majority of genes required for excitation-contraction coupling, the absence of Na_v_ and K_v_ channels in sponges precludes rapid fluctuations in membrane potential, at least by canonical means. Sponges do possess K_2P_ leak (Wells et al., 2012) and inward rectifying (Tompkins-Macdonald et al., 2009) K^+^ channels, which if expressed in contractile cells would establish negative resting membrane potentials. However, in fresh and sea water sponges from the genus *Microciona*, increasing external [K^+^], which diminishes the K^+^ membrane gradient and would depolarize cells, has no bearing on contraction (Prosser, 1967). However, contraction does depend on the presence of external cations, which presumably move across the cell membrane, albeit in a non-selective manner: External Na^+^ can be substituted with K^+^ or Li^+^, and Ca^2+^ can be replaced with Mg^2+^ or Sr^2+^. Notable is that cellular contractility appears highly atypical in this clade, in its dependence on both external Ca^2+^ and Mg^2+^, but perhaps more Mg^2+^, being roughly 5-fold more concentrated than Ca^2+^ in sea water. Instead, specimens from the genus *Euspongia* do show a selective dependency on external Na^+^ and Ca^2+^, and increasing external [K^+^] triggers marked and prolonged contraction consistent with depolarization-induced contractility (Pavans de Ceccatty, 1971). Nonetheless, these contraction events occur in the absence of measurable electrical impulses, and their slow and long-lasting kinetics make it unlikely that voltage-gated channels play a role.

The absence of gap junctions in sponges, combined with the absence of electrical signaling in cellular sponge species in general, indicate that contraction and its propagation from cell-to-cell occurs though much slower cellular pathways. Some have therefore speculated that contractile waves spread along sponge tissues by means of paracrine secretion, where incoming paracrine factors cause cells to both contract and to secrete (Leys and Meech, 2006). With respect to cellular contraction, its dependency on extracellular cations implies that ions move across the membrane, perhaps through ion channels, pumps and/or exchangers which are regulated by ligand-dependent receptors such as G-protein coupled receptors (GPCRs). Several “slow” GPCR pathways exist in muscle which dynamically regulate myosin light chain phosphorylation/dephosphorylation and hence contraction of actin/myosin filaments (Somlyo and Somlyo, 2003). In addition, some GPCR pathways directly regulate muscle contraction and tone through release of Ca^2+^ from internal stores and/or activation of other muscle effector kinases such as protein kinase C and Rho-associated protein kinase (Sanderson et al., 2008); in this context, membrane Ca^2+^ influx is thought necessary only for the replenishment of internal stores, and not for regulating contraction *per se*. Thus, contractile activity in the absence of fast electrical signaling is not unprecedented. Beyond pinacocytes and actinocytes, other sponge cells exhibit extensive motility, which depends on influx of extracellular calcium and could contribute to gross body movement via cumulative action of multiple cells (Lorenz et al., 1996).

#### Electrical signaling

Glass sponges (class Hexactinellida) are the only poriferan group known to exhibit electrical signaling, in the form of Ca^2+^ action potentials which travel through a multinucleated syncytium comprising the entire body (Leys and Mackie, 1997; Leys and Meech, 2006). Although some cells remain separate, they are nevertheless connected to the syncytium though cytoplasmic bridges (Mackie, 1981), making the entire glass sponge body one large electrically conductive system. Unlike cellular sponges, glass sponges are incapable of contracting, and as noted above, prevent particulates from entering their aquiferous systems by arresting choanocyte ciliary beating and the feeding current (Mackie, 1979; Lawn et al., 1981). The entire process, from stimulus onset to current cessation, occurs within roughly 20 s and can be triggered either spontaneously, in response to excessive sediment in the water, or via applied mechanical or electrical stimulation (Lawn et al., 1981; Mackie et al., 1983). External recording of stimulated tissue reveals a biphasic action potential, with a depolarizing inward cation current preceding a repolarizing current presumably carried by efflux of K^+^ ions (Leys and Mackie, 1997; Leys et al., 1999). Voltage-gated Ca^2+^ channels are thought to mediate action potential depolarization, since reduction of external [Na^+^] to 25% of physiological levels only minimally affects the action potential, whereas application of Ca_v_ channel blockers Co^2+^ and Mn^2+^ and Nimodipine significantly disrupt it (Leys et al., 1999). Given the loss of Na_v_2 channels in sponges, the single Ca_v_ channel found in the sponge genome is a viable molecular candidate for driving the glass sponge action potential. Not clear is how repolarization takes place in the absence of K_v_ channels. Application of K_v_ channel blocker tetraethylammonium (TEA) delays and diminishes the amplitude of the action potential, but it does not specifically prolong the depolarization phase, which would be expected if blocking a repolarizing K_v_ channel current. Given the slow kinetics of the action potential, it might be that repolarization involves slower K^+^ conductances, such as K_2P_ or K_IR_ channels. Furthermore, contributions to repolarization might be attributed to accumulated inactivation of the voltage-gated Ca^2+^ channel, consistent with the observed refractory period of the sponge action potential of roughly 29 s (Leys et al., 1999).

Also not clear is how propagating Ca^2+^ action potentials lead to arrest of choanocyte ciliary beating. Based on the observed involvement of calcium channels in regulating *Chlamydomonas* and protozoan ciliary beating, Ca_v_ channels and Ca^2+^ influx have been suggested to play a role (Leys and Meech, 2006), however, their localization to choanocytes, and the Ca^2+^-sensitivity of choanocyte-driven feeding current, have yet to be explored. Likewise, involvement of the sponge Ca_v_ channel in altered ciliary beating of larvae in response to light stimulation has not been explored, where perhaps the mechanisms underlying both choanocyte ciliary arrest and larval ciliary switch use overlapping mechanisms.

### Ctenophora

**Figure 6:**
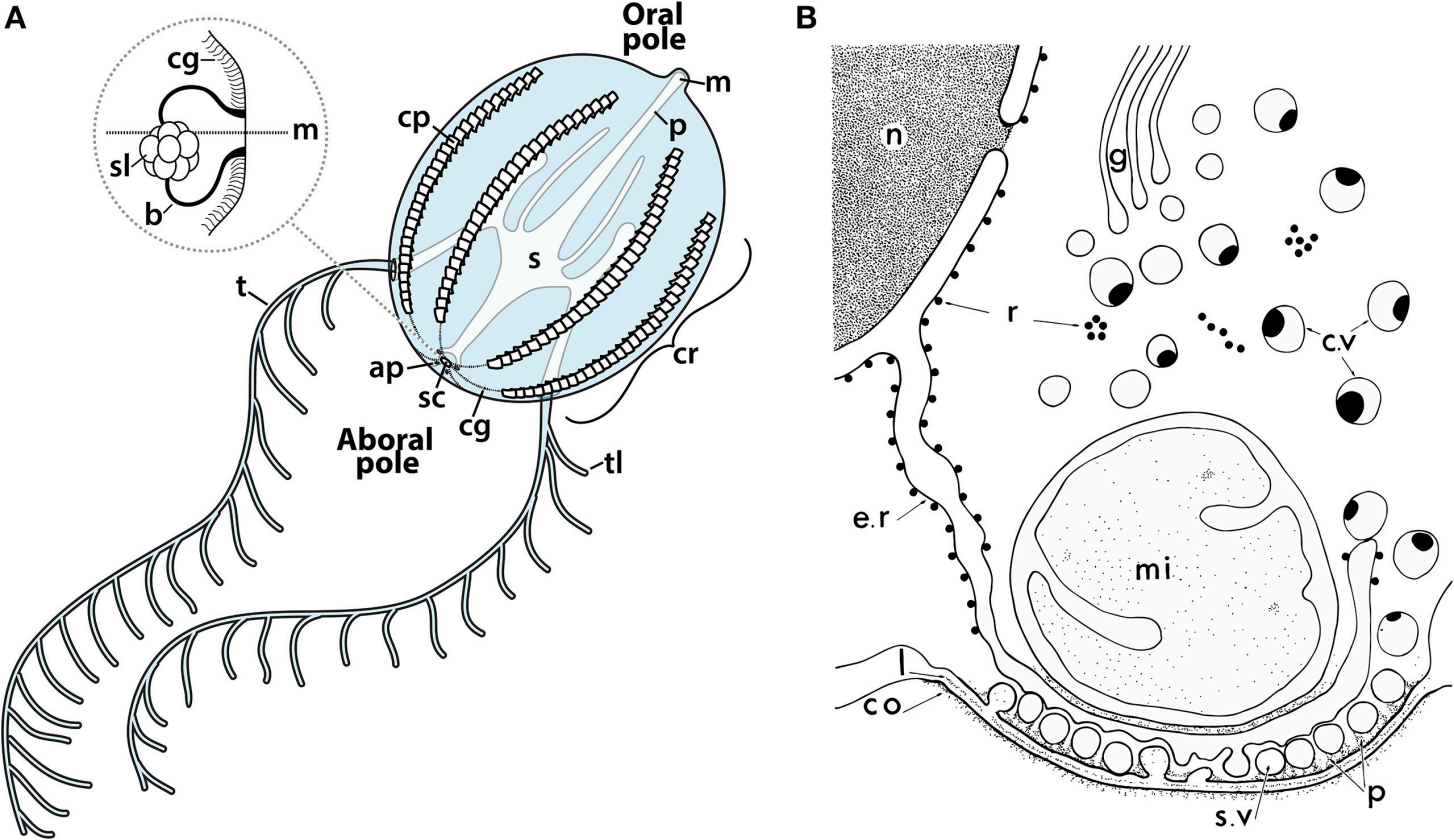
**(A)** Illustration of a cydippid ctenophore, showing the oral and aboral poles bearing the mouth (m) and statocyst (sc), respectively. Ctenophores possess eight comb rows (cr), each made up of a series of comb plates (cp) which beat in the oral-aboral direction during forward swimming, or aboral-oral direction during reverse and rotational swimming. Geotactic control of comb row beating occurs via signal transduction from the statocyst, a ciliated gravitometric organ, to the beginning of each comb row via ciliated grooves (cg). Tentacles (t) and tentilla (tl) bear colloblasts, laden with adhesive granules used for prey capture; injested food enters the mouth into the pharynx (p), and eventually the stomach (s) and digestive system. Inset: Side view of two balancers (b) of the statocyst of an animal in the horizontal position, connected at their tips to the statolith (sl). Weight from the statolith mechanically deflects the balancers either toward or away from the midline (m), mechanically activating the beating of balancer cilia; these then activate waves of beating in the ciliated grooves (cg) which propagate to the comb rows. **(B)** Illustration of the pre-synaptic triad of ctenophore synapses, consisting of rows of synaptic vesicles (sv) arranged along the membrane, adjacent to a finger-like projection of smooth endoplasmic reticulum, which lacks ribosomes (r) of the rough endoplasmic reticulum, and one or several large mitochondria (mi). n, nucleus; g, Golgi, c.v, cytoplasmic vesicles; co, post-synaptic dense coat; p, pre-synaptic dense projections. Reprinted with permission from Hernandez-Nicaise (1973a).

Ctenophores, or comb jellies, were classically grouped with cnidarians in the clade Coelenterata (Leuckart, 1848), bearing similar morphological characters to jellyfish. Current phylogenetic studies however provide compelling evidence against the monophyly Coelenterata (see above), where instead, ctenophores might represent most early-diverging animals, separated from cnidarians by Porifera and Placozoa. Accordingly, detailed comparison of morphological, physiological, and gene content characters between ctenophores and cnidarians points to a deep and ancient divergence. For example, ctenophores exhibit complex bi-radial body patterning acquired through a distinct developmental program (Chun, 1880; Driesch and Morgan, 1895; Freeman, 1977; Fischer et al., 2014), yet they lack key homologs for genes involved in body patterning and development crucial in Cnidaria and Bilateria, including homeobox genes of the Hox and ParaHox classes (Ryan et al., 2010) and major components of the Notch and Hedgehog cell signaling pathways (Walton et al., 2006; Gazave et al., 2009; Ingham et al., 2011; Ryan et al., 2013; Moroz et al., 2014). Both ctenophores and cnidarians possess diffuse “polygonal” nerve nets. However, nodes of the ctenophore net are connected by anastomosed (bundled) axon/neurite projections (Jager et al., 2011), while in cnidarians they are connected by only single neurites (Satterlie, 2011). The ultrastructure of the ctenophore chemical synapse is also different, consisting of a unique “pre-synaptic triad” arrangement of a row of membrane-lined vesicles separated from closely apposed mitochondria by a thin finger-like projection of smooth endoplasmic reticulum (SER)(Horridge and Mackay, 1964; Hernandez-Nicaise, 1973a) (Figure 6A). Some reciprocal synapses in cnidaria also consist of membrane-lined vesicles and an adjacent cisternal structure (Ryan and Chiodin, 2015), however, close apposition of mitochondria is not ubiquitous, and vesicles are documented to not bud off of the cisternal structure as they appear to do in ctenophores (Hernandez-Nicaise, 1973a; Anderson and Grünert, 1988). Synaptic divergence is also evident in corresponding chemical lexicons used for synaptic transmission, where ctenophores lack key genes for the biosynthesis and transport of neurotransmitters in cnidarians/bilaterians; instead, ctenophores exhibit unique gene duplications suggesting that synaptic transmission relies on expanded peptidergic and glutamatergic signaling systems (Moroz et al., 2014; Moroz and Kohn, 2015, 2016). Fundamental distinctions between ctenophores and cnidarians also extend to the tentacles and muscle. Both use tentacles for prey capture, however, jellyfish tentacles bear stinging cnidocytes which are unique to the Cnidaria (see above), whereas ctenophore tentacles contain colloblasts (a.k.a. glue cells), unique to ctenophores and lined with granules of sticky substances that burst open upon contact to ensnare prey by adhesion (Franc, 1978). Ctenophores possess smooth (Hernandez-Nicaise, 1991) and striated (Hertwig and Hertwig, 1879; Mackie et al., 1988) muscle cells derived from a “mesoderm” cell layer (Martindale, 2005), thought to be absent in Cnidaria (Martindale et al., 2004; Burton, 2008). Thus, ctenophores appear to resemble bilaterians, having mesoderm-derived muscle cells, while cnidarians independently evolved striated muscle (Steinmetz et al., 2012) and likely their epithelium-derived muscle sheets involved in bell contraction (i.e., the myopeithelium). However, ctenophore genomes have a major deficiency in genes required for mesoderm development in bilaterians (Ryan et al., 2013; Moroz et al., 2014), arguing against ctenophore-bilaterian homology and suggesting that ctenophores also independently evolved muscle (and the mesoderm).

One of the most distinguishing features of ctenophores are their comb rows, eight longitudinal strips of beating ciliary paddles (a.k.a. comb plates or ctenes) used for swimming, that run from the statocyst (aboral pole) to the mouth of the animals (oral pole) (Tamm, 2014a) (Figure 6A), and diffract light to generate the striking and characteristic rainbow coloration of comb jellies. Each comb plate is made up of thousands of amalgamated cilia which beat in unison with asymmetric power and recovery strokes. Ctenophore locomotion thus results from propagating waves of comb plate power strokes that travel along the comb rows in either an aboral-oral direction (i.e., during forward swimming), or an oral-aboral direction (during reverse swimming) (Tamm and Tamm, 1981). Comb row beating is highly regulated by neuronal and non-neuronal integrative pathways (Tamm, 2014a), and as a result, ctenophores exhibit complex swimming behaviors including positive and negative geotaxis (Horridge, 1971; Tamm, 1980, 1982; Lowe, 1997), feeding behavior (Swanberg, 1974; Tamm and Moss, 1985; Moss and Tamm, 1986, 1987, 1993; Haddock, 2007), and stimulus responses such as pausing (Göthlin, 1920) and escape reverse/fast-forward swimming (Mackie et al., 1992; Kreps et al., 1997; Tamm, 2014a). Besides comb plates, motile cilia play a major role in ctenophore biology, including toothed macrocilia located inside the mouths of predatory beroid species, used for biting and engulfing prey (Swanberg, 1974; Tamm and Tamm, 1988), and balancer cilia of the statocyst (Figure 6A), a gravity receptor located at the aboral pole, where four balancers transduce angular body displacement to changes in comb row beat frequency during geotactic behavior (Chun, 1880; Tamm, 1982, 2014a,b, 2015). Notably, although locomotion is largely mediated by comb rows, ctenophores employ muscles for numerous motile behaviors, in particular with respect to feeding, such as the dramatic engulfing of prey by beroids (Swanberg, 1974; Bilbaut et al., 1988a,b; Haddock, 2007), the retraction of tentacles upon prey capture (Hertwig and Hertwig, 1879; Mackie et al., 1988), and the movement of oral structures such as lobes and auricles of lobate species to direct food into the mouth (Tamm, 1982, 2014a; Larson, 1988).

Voltage-activated Ca^2+^ currents are implicated in numerous aspects of ctenophore biology, including electrical signaling, muscle contraction, ciliary reversal of comb rows, beat frequency of balancer cilia of the statocyst, and activation of macrocilia in the beroid mouth. As noted above, ctenophores possess a single Ca_v_ channel phylogenetically similar to Ca_v_2 types (Figure 4), as well as two Na_v_2 channels which are likely Ca^2+^ permeable if not Ca^2+^ selective. Below, we discuss some experimental observations that implicate Ca_v_ and/or Na_v_2 channels in ciliary and muscle function in ctenophores, and discuss their potential involvement in the less understood processes of neural excitation and synaptic transmission.

#### Electrical signaling and synaptic transmission

Phylogenetic analyses suggest that ctenophores are the most basal animals (Dunn et al., 2008; Ryan et al., 2013; Moroz et al., 2014; Whelan et al., 2015), leading to the controversial hypothesis that they independently evolved a nervous system (Marlow and Arendt, 2014; Moroz et al., 2014; Moroz and Kohn, 2015, 2016; Ryan and Chiodin, 2015). The most identifiable feature of the nervous system are synapses, defined by the presence of pre-synaptic active zones, connected across the synaptic cleft to electron-dense post-synaptic densities (Heuser and Reese, 1977). Genome sequencing has revealed that the vast majority of genes involved in synapse formation and function are present in ctenophores (Ryan et al., 2013; Moroz et al., 2014; Moroz and Kohn, 2015), animals which lack synapses [sponges (Srivastava et al., 2010) and placozoans (Srivastava et al., 2008)], and even unicellular organisms that pre-date animals (King et al., 2008; Fairclough et al., 2013). Thus, the mere presence or absence of select synaptic genes, in particular those with more generalized functions in exocytosis not specific to synapses, is not enough to confirm or refute the independent evolution hypothesis, since most of these genes were present before the ctenophore divergence. Resolving this issue will require extensive molecular comparative analyses of nervous system development and function.

The ctenophore nervous system consists of two distinct nerve nets (Jager et al., 2011), as well as separate neural structures that innervate peripheral structures such as the tentacles, comb rows and mouth (Hernandez-Nicaise, 1973b, 1991; Mackie et al., 1992; Tamm and Tamm, 1995; Jager et al., 2011). Giant axons have been documented running under the comb rows of select species, which synapse onto comb plate polster cells and are believed to alter ciliary beating during escape swimming (Mackie et al., 1992). Here, the large-diameter axons are considered an adaptation to increase action potential velocity, with speeds greater than 0.5 ms^−1^ comparable to the 1.4 ms^−1^ of giant axons of the jellyfish *A. digitale* escape system (Mackie and Meech, 1985). Large diameter axons have also been documented in the mouths of beroids, which synapse onto smooth muscle and adhesive epithelial cells to coordinate swallowing of prey and the subsequent tight closure of the mouth (Tamm and Tamm, 1995). Thus, fast neuronal signaling is certainly confirmed in ctenophores, but the mechanisms by which these signals are transduced across the synaptic cleft remains a mystery. Indeed, the atypical ultrastructure of the ctenophore synapse suggests that the underlying mechanisms for synaptic transmission might be inherently different from other animals. Most notable is that in ctenophores pre-synaptic vesicles appear to bud off from the SER (or perhaps fuse with it), suggesting that vesicles are derived independently of the Golgi network and pre-synaptic endosomes, as in bilaterian synapses (Heuser and Reese, 1973; Jahn and Fasshauer, 2012). If this proves to be true, it would represent a striking fundamental difference in synapse organization and function consistent with independent evolution. By extension, this arrangement would provide ER-derived neuropeptides direct access to pre-synaptic vesicles, consistent with the notion that ctenophore synapses rely heavily on neuropeptides as transmitters (Jager et al., 2011; Moroz et al., 2014; Moroz and Kohn, 2016).

The presence of both SER and large mitochondria within nanometer proximity of the putative vesicle release sites suggests that Ca^2+^ plays a role in ctenophore synaptic exocytosis, where the two organellar systems are poised to act as sources or sinks for Ca^2+^ ions (Clapham, 2007). Unfortunately, only a few examples of electrophysiologically-recorded synaptic potentials are available for ctenophores (Moss and Tamm, 1987; Meech, 2015), and no experimental evidence is available describing the involvement of the single Ca_v_2-like channel or other Ca^2+^ channels in vesicle exocytosis. In bilaterian synapses, Ca^2+^ influx through Ca_v_2 channels is required to activate Ca^2+^-sensitive exocytotic machinery, and this is achieved by close apposition of the channels with docked vesicles. As noted above, some interactions with scaffolding proteins that help tether Ca_v_2 channels at the synapse appear to have deep ancestry, occurring in both protostome and deuterostome bilaterians (e.g., Rim-1, Mint1, and CASK). If these interactions extend to both cnidarians and ctenophores, this would strengthen the argument for the single origin of the nervous system hypothesis. Instead, the obligate close apposition of SER and mitochondria to what appear to be docked vesicles in the ctenophore synapse might indicate that Ca_v_ channel nanodomain coupling is circumvented in lieu of calcium-induced calcium release from the SER, a mechanism for synaptic transmission that would be highly atypical. Clearly, this area of research warrants further study, to explain homology or convergence between synapses in ctenophores and those in cnidarians/bilaterians.

#### Muscle contraction

The most detailed electrophysiological records of membrane ion currents in ctenophores come from enzymatically-isolated smooth muscle cells. These cells are multinucleated and can be massive (upwards of 6 cm long and 40 μm in diameter), and are innervated by nerve net neurons at neuromuscular junctions bearing the presynaptic triad arrangement (Hernandez-Nicaise, 1973a). Isolated muscle cells from the cydippid *Pleurobrachia bachei* conduct action potentials and contract in response to external perfusion of L-glutamate, as less so L-aspartate, but not to other transmitters such as GABA, histamine and acetylcholine (Moroz et al., 2014). Thus, ctenophore neuromuscular junctions are proposed to be glutamatergic, although the various ionotropic glutamate receptors present in the genome have yet to be localized to muscle synapses. Intracellular voltage clamp experiments reveal diverse pools of ion channels in ctenophore smooth muscle, which generate distinct action potential waveforms in different cell types. In *Mnemiopsis* giant muscle cells, which project from the statocyst to the mouth and auricles (Anderson, 1984; Hernandez-Nicaise et al., 1984), the depolarization phase of the action potential is driven by at least two distinct voltage-gated channels permeable to Ca^2+^ and Na^2+^ (but more selective for Ca^2+^), both high voltage activated and sensitive to Ca_v_ channel blockers Cd^2+^, Co^2+^ and dihydropyridines verapamil and methoxyverapamil (Anderson, 1984; Dubas et al., 1988). One notable distinction was their kinetics, with one channel bearing fast inactivation producing fast, transient Ca^2+^ currents, and the other much slower inactivation, producing slow, long-lasting currents (Dubas et al., 1988). Similar depolarizing Ca^2+^/Na^+^ currents were reported for the action potential of giant muscle cells isolated from *B. ovata* (Hernandez-Nicaise et al., 1980, 1982; Bilbaut et al., 1988a,b). For both species, Ca^2+^ influx through the channels seems required for contraction, since removal of external Ca^2+^ or pharmacological disruption abrogates muscle action potentials and contractions (Hernandez-Nicaise et al., 1980; Anderson, 1984; Bilbaut et al., 1988a,b; Dubas et al., 1988; Cario et al., 1996). Unfortunately, while the available data provides convincing evidence for the existence of distinct voltage-gated Ca^2+^ channels present in ctenophore smooth muscle, little can be said about the specific channel types at play. The three most likely candidates, the single Ca_v_2 and two Na_v_2 channels, are expected to be highly divergent from their homologs in other animals, and their specific pharmacological, ion selectivity and biophysical properties are completely unknown. Further confounding the matter is that currents recorded from a cloned Na_v_2 channel (from honeybee) are sensitive to Cd^2+^ but not dihydropyridines (Gosselin-Badaroudine et al., 2016), while *in situ* currents recorded from cnidarian neurons are sensitive to both Cd^2+^ and dihydropyridines (Anderson, 1987; Spafford et al., 1996). Ca^2+^ vs. Na^+^ selectivity is also not a good marker for channel identity, since it can be quite variable, a fact made evident by the T-type channel from snail *L. stagnalis* which becomes highly Na^+^ permeable via alternative splicing in the domain II P-loop region, without altering its Ca^2+^ selectivity filter motif of EEDD (Senatore et al., 2014). Nonetheless, it is worth noting that ctenophores are the only animals with *bona fide* muscle cells lacking Ca_v_1 channels, the main drivers for excitation-contraction coupling in other animals. In this regard, and in accordance with the proposed independent evolution of ctenophore muscle (Ryan et al., 2013; Moroz et al., 2014), smooth muscle cells seem to depend on somewhat atypical depolarizing conductances for excitation-contraction coupling.

In *B. ovata*, movements of the mouth and pharynx during swallowing of prey are encoded by different conductance/contractile profiles of distinct muscle cell types, in lieu of a complex nervous system capable of sophisticated temporal and spatial synaptic outputs (Bilbaut et al., 1988a,b). Radial smooth muscle cells, which span the mesoglea and are anchored in the outer epidermis (ectoderm) and pharyngeal endoderm (Hernandez-Nicaise et al., 1980), exhibit narrow, transient action potentials required in bursts for contraction to occur. Instead, longitudinal muscles running along the ectoderm exhibit longer lasting action potentials, each capable of causing contraction. The differences in action potential waveforms are attributed to different repolarizing conductances: Radial fibers bear pronounced, rapidly activating K^+^ currents, while longitudinal fibers bear a slow Ca^2+^-activated K^+^ current (K_Ca_), and a transient, voltage-sensitive K^+^ current. Worth noting is that the functional coupling of voltage-gated Ca^2+^ channels with Ca^2+^-sensitive K^+^ channels (e.g., K_Ca_ channels such as BK and SK), also documented in *Mnemiopsis* smooth muscle (Anderson, 1984), predates animals, observed in protists (Valentine et al., 2012) and dinoflagellates (Pozdnyakov and Skarlato, 2015). In vertebrates, both BK and SK K_Ca_ channels physically and functionally couple with Ca_v_1, Ca_v_2, and Ca_v_3 channels, presumably to overcome the limited diffusion range of Ca^2+^ ions in the cytoplasm (Clapham, 2007; Guéguinou et al., 2014). This coupling is observed in neurons which undergo spike frequency adaptation, where trains of action potentials accumulate more and more cytoplasmic Ca^2+^ and K_Ca_ channel activation, leading to a slowing down of action potential frequency and an eventual disruption of the action potential spike train (Yarom et al., 1985). In accordance, longitudinal but not radial muscle in *Beroe* exhibit spike frequency adaptation and eventual cessation of induced spikes. As noted by the authors, the difference in muscle properties would permit bursts of synaptic inputs from the nerve net to cause short lived contractions of longitudinal fibers, while causing facilitating, long lasting contractions of radial fibers, permitting more complex movement of the mouth and pharynx (Bilbaut et al., 1988a).

It is unknown whether membrane Ca^2+^ influx alone activates contractile myofilaments, or whether internal stores from the sarcoplasmic reticulum or mitochondria contribute via CICR or some other mechanism. Notably, the sarcoplasmic reticulum (SR) is diminished compared to other smooth muscle cells, making up less than 1% of the total cell volume, and that the plasma membrane (sarcolemma) lacks typical invaginations and appositions with the SR (Hernandez-Nicaise and Amsellem, 1980; Hernandez-Nicaise et al., 1980, 1984), both of which serve to enhance the CICR process. Furthermore, in larger cells, the sarcolemma can be as far as 2000 nm away from the SR and mitochondria(Hernandez-Nicaise et al., 1980). Considering the observed range of Ca_v_ channel cytoplasmic Ca^2+^ plumes of roughly 100 nm, even in elevated external [Ca^2+^] (Weber et al., 2010), this separation seems rather imposing. Nevertheless, ATP-hydrolyzing enzymes that would shuffle Ca^2+^ ions into the SR and mitochondria, as well as out of the cell through the sarcolemma, have been detected (Cario et al., 1996), and Ca^2+^ fluorescence experiments reveal that action potentials trigger Ca^2+^ release from internal stores (Cario et al., 1995a,b). Thus, CICR likely takes place in ctenophore smooth muscle at least to some degree. Interestingly, all smooth muscle cells observed possess extracellular axonemes (i.e., bare cilia), which run along the lengths of the muscle fibers nestled within circumferential invaginations of the sarcolemma (Tamm and Tamm, 1989). The mechanism by which these structures are formed, and their function, remains a mystery. Given the propensity of cilia to bind Ca^2+^, they have been proposed to act as external Ca^2+^ sources/sinks for excitation-contraction coupling (Tamm, 2014a).

Ctenophores also possess striated muscle. Cydippid specimens of the genus *Euplokamis* possess tentacles with specialized repeating side branches (tentilla) which extend and retract to lure and capture prey with their colloblasts (Chun, 1880). Tentilla are heavily innervated, and exhibit complex localized sensory integration, where tactile or electrical stimulation can activate rapid extension of single tentilla through contraction of bundled, long striated muscle cells running along their lengths; subsequent retraction and coiling is thought to occur passively, though the elastic properties of the underlying tissue (Mackie et al., 1988). Activation of this muscle is clearly through excitation, since depolarization of the membrane with high external [K^+^] or electrical stimulation causes contraction (Mackie et al., 1988). *In vivo*, excitation-contraction coupling is likely elicited at neuromuscular junctions located along the outside of the bundled fibers, with few mitochondria indicating low energy expenditure as expected given the infrequent use of the tentilla for prey capture. Repeating striations in the muscle are clearly evident, bearing distinct Z, I, and A bands characteristic of striated muscle, but they lack H bands. Ctenophores lack key genes associated with striated muscle formation and function, including those involved in Z-disc formation (Steinmetz et al., 2012). Thus, striated muscle in *Euplokamis* might represent a third case of independently evolved striated muscle, along with bilateria and cnidaria. Like giant smooth muscle cells of *Beroe* and *Mnemiopsis, Euplokamis* striated muscle cells lack extensive SR at their center, as well as any obvious transverse tubular systems. However, they do possess a second arrangement of SR located within a few hundreds on nanometers from the sarcolemma. The involvement of membrane Ca^2+^ influx through voltage-gated calcium channels and CICR in contraction has not yet been examined, however the presence of synapses, the rapid speed of contraction, and electrical activation of these striated muscle cells imply these processes do occur.

#### Balancer cilia in the statocyst

On their own, comb plates are static and require external inputs to initiate beating (Sleigh, 1974; Tamm, 1980). A major source of activation arrives from tracks of ciliated epidermal cells, called ciliated grooves, which transduce gravitational signals from the statocyst to the first comb plate of each row (Chun, 1880) (Figure 6A). The four balancers of the statocyst each consist of compound cilia, which at their tips support a conglomerated mass of living cells called the statolith, much like four legs supporting a table. Angular body displacement causes the statolith to exert differential gravitational force on each of the balancers, altering their respective beating frequency according to the angle of force exertion (Tamm, 1982). The balancers act as pacemakers for the comb rows via mechanical coordination (Tamm, 1982) (Figure 6A). During negative phototactic swimming, when ctenophores seek swim to the surface mouth facing up, tilting toward the horizontal causes balancers (and hence comb rows) below the midline to beat more frequently than ones above, while the opposite occurs during downward, mouth down (i.e., positive geotactic) swimming. Once the animals are vertical, all eight comb rows beat at a similar frequency (Tamm, 1980, 1982). Interestingly, evidence suggests that deflection-induced changes in balancer beating requires membrane excitation and Ca^2+^ influx of through voltage-gated channels, since removal of external Ca^2+^, or application of non-specific calcium channel blockers Co^2+^ and Ni^2+^, disrupt deflection-induced responses of the balancers (Lowe, 1997). Furthermore, chemical depolarization of isolated balancers (via increasing external [K^+^]) directly increases beating frequency independent of mechanical stimulation, but only in the presence of external Ca^2+^ specifically at the base of the cilium. Thus, a proposed model for statolith activation of balancer beating is that deflections activate cationic stretch-receptors at the cell membrane, which in turn activate voltage-gated calcium channels at the base of the cilium. Ca^2+^ influx through these channels then activates ciliary beating. Notably, such an arrangement appears inconsistent with sperm flagella, where hyperactivation (i.e., an alteration of ciliary waveform) depends on the voltage-gated Ca^2+^ channel CatSper localized along the length of the cilium, not at the base. Noted above, in *Chlamydononas*, a Ca_v_ channel homolog dubbed CAV2 causes flagellar waveform change, and is also localized strictly to the distal regions of the cilia. A notable distinction between ctenophore balancers and these two other systems is that in balancers, Ca^2+^ influx at the base serves to *activate* ciliary beating, whereas in sperm and *Chlamydomonas*, distal Ca^2+^ influx along the ciliary membrane serves to *alter* the waveform of ciliary beating, either by increasing asymmetry of the flagellar waveform (sperm), or by increasing waveform symmetry (*Chlamydomonas*). Consistent with this distinction, activation of distal voltage-gated calcium channels in comb plate cilia leads to reversal of beating during backward swimming and feeding behavior, whereas voltage-gated Ca^2+^ channels located at the base of beroid macrocilia activate beating. These similarities prompted Tamm to propose conserved mechanisms for voltage-gated Ca^2+^ channel regulation of ciliary beating, where channels located along the length of the cilia influence “reprogramming” responses of the ciliary waveform (e.g., reversal, waveform changes), whereas those at the base of the cilia influence “on-off” responses (e.g., activation, arrest, or increase in beating frequency) (Tamm, 1994, 2014a).

The propensity of ctenophores to switch between upward swimming (negative geotaxis) and downward swimming (positive geotaxis) is referred to as their “mood,” and is thought to be regulated by neural inputs to balancer cells from various sensory modalities, including those tuned to water disturbances and hydrostatic pressure (Tamm, 1982, 2014a; Lowe, 1997). Electron microscopy of the statocyst and surrounding areas reveals neurites which synapse onto balancer cells(Tamm, 1982; Hernandez-Nicaise, 1991). Furthermore, intact larvae and dissected statocysts from *M. leidyi* and *Pleurobrachia pileus* respond to ectopic electrical stimulation and membrane depolarization with high external [K^+^] by switching between geotactic states (Lowe, 1997), consistent with regulation by electrical signaling. Mechanistically, a switch in mood/geotactic state requires that the same deflectional forces acting on the balancer cilia produce opposite effects on their beating frequency at different times. Nonetheless, regardless of geotactic sign, ciliary deflection in the appropriate direction causes Ca^2+^ influx and increased beating. Thus, the mechanisms by which putative synaptic inputs alter geotactic mood in the balancers are likely independent of the stretch-activated channels and voltage-gated Ca^2+^ channels. Finally, ctenophores are able to override geotactic behavior and exhibit different types of swimming, such as horizontal, feeding, and reverse escape. The mechanisms by which this takes place are not known (Tamm, 2014a); neural inputs to the balancers, ciliated grooves, and/or comb plates is a possiblility.

#### Comb plate cilia and macrocilia

In *Euplokamis*, electrical stimulation near the mouth causes a temporary reversal of comb plate beating and thus reverse swimming, presumably via nerve impulses from giant axons running under the comb plates which synapse onto polster cells (Mackie et al., 1992). Instead, stimulation at the aboral end causes increased ciliary beating for fast-forward swimming, which based on experiments in *Pleurobrachia*, might occur via nerve inputs downstream of the statocyst perhaps at the ciliated grooves or the aboral-most comb plates (Tamm, 1982). Similar fast-forward and reverse swimming responses are observed for *Mnemiopsis* upon contact with a jellyfish predator (Kreps et al., 1997). Unilateral reversal of comb plate beating is also observed during cydippid feeding (Tamm and Moss, 1985), where in *Pleurobrachia*, comb rows flanking tentacles with ensnared prey reverse, leading to rotation of the animal such that the appropriate tentacle bearing food approaches the mouth. Here, direct electrical stimulation of a single tentacle or its adjacent body surface causes comb plate reversal, through an apparently bilateral conduction pathway innervating only the four ipsilateral comb rows of that tentacle (Moss and Tamm, 1993). The mechanism for comb plate reversal is thought to occur directly in comb plate polster cells, where intracellular recording at the cell soma revealed that neural stimulus-induced synaptic potentials give rise Ca^2+^-dependent action potentials, leading to ciliary reversal (Moss and Tamm, 1986). Extracellular recording and Ca^2+^ imaging of comb plate cilia revealed that action potentials propagate from base to tip of the cilium (Tamm and Terasaki, 1994), and could be abrogated by application of inorganic calcium channel blockers (Moss and Tamm, 1987). Thus, similar to *Chlamydomonas* CAV2, a voltage-gated Ca^2+^ channel distributed along the ciliary membrane mediates motor responses. It will be interesting if this channel turns out to be Ca_v_2, suggesting an ancient and conserved coupling of Ca_v_ channels types with ciliary reversal.

Finally, giant “toothed” macrocilia located inside the mouth of predatory beroid species receive synaptic input from a giant axon nerve net (Tamm and Tamm, 1985, 1995), whose excitatory inputs activate beating from an otherwise intermittent/quiescent state during engulfing of prey (Tamm, 1983, 1988a). The macrociliary power stroke is directed into the body cavity, helping to draw or macerate prey into the stomach (Swanberg, 1974). Notably, both semi-intact preparations of macrocilia, as well as isolated macrociliary cells, become activated in response to depolarization with high external [K^+^], but only in the presence of external Ca^2+^ which need only be applied by perfusion to the base of the cilium (Tamm, 1988a,b). Furthermore, application of non-selective calcium channel blockers prevents macrociliary activation (Tamm, 1988a). Indeed, all of the data is consistent with a model where synaptic inputs depolarize the membrane to activate voltage-gated Ca^2+^ channels strictly at the base of the cilia to initiate beating (Tamm, 2014a). Thus, either differential localization of the same voltage-gated calcium channel used to reverse beating of comb plate cilia, or a different channel altogether (perhaps also used at the base of balancers), plays the role of activating beating of macrocilia, consistent with Tamm's hypothesis on localization-dependent “on-off” vs. “reprogramming” function of ciliary calcium channels.

## Conclusions

Recent studies suggest that four domain P-loop channels, which include Ca_v_ and Na_v_ channels, evolved as Ca^2+^-selective channels with selectivity filter motifs enriched in glutamate and aspartate residues, producing high-affinity binding sites for Ca^2+^ in the pore (Liebeskind et al., 2011; Moran et al., 2015). Later, in bilaterians and cnidarians, Na^+^ selective channels emerged, allowing for separation of electrogenic depolarizing Na^+^ currents from Ca^2+^ signaling (Barzilai et al., 2012). In general, Ca_v_ and Ca^2+^-selective Na_v_2 channels provide excitable cells with a means of exerting rapid and transient changes in cellular proteins through Ca^2+^-dependent alterations in their structure and complexing. An array of such proteins have evolved, including Ca^2+^-sensitive ion channels, components of the exocytotic machinery, proteins involved in control of ciliary beating, and signaling proteins. Key to these functional associations is proximity; Ca^2+^ is actively sequestered and extruded from the cytoplasm, so Ca_v_ channels need to be positioned close to their cytoplasmic partners, and often physically couple with them either directly or through protein intermediaries. In the organismal lineages leading to Metazoa, different types of Ca_v_ channels evolved with distinct voltage dependencies, kinetics of activation and inactivation, and Ca^2+^ selectivity, most distinguishable between high voltage-activated Ca_v_1 and Ca_v_2 type channels and low voltage activated Ca_v_3 type channels. In the nervous system, this fundamental distinction means that Ca_v_3 channels are best suited for helping neurons decide when to fire action potentials, while Ca_v_1 and Ca_v_2 channels are brought in as effectors once the decision has been made. This bifurcation appears quite ancient, dating at least as far back as the divergence between choanoflagellates and metazoans (Liebeskind et al., 2011; Barzilai et al., 2012; Fairclough et al., 2013; Moran and Zakon, 2014). Cnidarans are the most basal lineage of animals to have a nervous system and possess all three types of Ca_v_ channels, which interestingly, are also present in *Trichoplax* which lacks a nervous system. More basal ctenophores and sponges only have a single Ca_v_ channel, where the ctenophore channel is phylogentically more similar to Ca_v_2 channels, and the sponge to Ca_v_1/2 or Ca_v_1 channels. In cnidaria and ctenophora, there are some interesting parallels in neuromuscular physiology compared to bilaterians; it will be interesting whether homologous functional and proteomic associations of Ca_v_ channels occur in these basal animals to account for these similarities. In Placozoa and Porifera, the lack of nervous systems begs the questions: What functions do “nervous system” genes serve in the absence of neurons and muscle? To what extent are the necessary protein complexes present, and what key elements do they lack that account for their absence of synapses?

The evolution of sophisticated processes involving Ca_v_ channels, as observed in neurons (excitation-transcription coupling), at the pre-synaptic terminal (excitation-secretion coupling), in muscle (excitation-contraction coupling) and in cilia (alteration of ciliary beating), might have involved innovations in cellular co-expression and subcellular complexing with other proteins. This, combined with the distinguishing ion conduction properties of the different Ca_v_ channels types, created a rich repertoire of modular interactions which could be deployed in different contexts to bring about desired cellular outputs. Not clear is whether the intrinsic properties and functional/proteomic associations of Ca_v_ channels, essential for nervous system function, largely predate the nervous system, or where extensively “tweaked” along the way. In the case of Ca_v_3 channels, intrinsic properties appear highly conserved, where the homolog from *Trichoplax* bears striking biophysical resemblance to human orthologs. Further comparative studies, evaluating the electrophysiological properties and proteomic interactions of Ca_v_ channels in early-diverging animals, is poised to provide valuable and interesting insights on animal evolution.

## Author contributions

AS wrote the initial draft of the manuscript. AS, HR, and PL revised the manuscript and generated the analyses and figures.

## Funding

Funding support was provided by NSERC Discovery (RGPIN-2016-06023) and CFI grants (CFI Project 35297), and University of Toronto startup funds to AS, and an NSERC USRA to PL.

### Conflict of interest statement

The authors declare that the research was conducted in the absence of any commercial or financial relationships that could be construed as a potential conflict of interest.
